# Fostering innovation: Experimental evidence on the effectiveness of behavioral interventions

**DOI:** 10.1371/journal.pone.0276463

**Published:** 2022-10-19

**Authors:** Elisa Matthewes, Anis Nassar, Christian Zihlmann

**Affiliations:** 1 Department of Economics, University of Fribourg, Fribourg, Switzerland; 2 Institute for Applied Data Science and Finance, Berne University of Applied Sciences, Berne, Switzerland; Universitat Jaume I Departament d’Economia, SPAIN

## Abstract

We experimentally investigate an intervention that ought to motivate innovative behavior by changing risk perceptions. Participants run a virtual lemonade stand and face a trade-off between exploiting a known strategy and exploring untested approaches. Innovation through testing new approaches comes along with a risk of failure because participants are compensated based on the profits generated by their virtual business. We test whether we can draw attention away from this risk by implementing a salience mechanism, which ought to focus participants on the input rather than the outcome of the innovative process. However, we find that this intervention is not effective in motivating innovative behavior—rather, it jeopardizes innovation. We discuss potential behavioral channels and encourage further research of risk salience as a tool to foster innovation. Our pre-registered study highlights the importance of evaluating interventions before implementation, as even carefully designed interventions may turn out to be ineffective or even backfire.

## Introduction

Innovation is required in many contemporary jobs and can drive firm performance and firm survival [1, 2]. Stimulating innovation is thus one of the main concerns of CEOs [3]. In this article, we test a behavioral intervention designed to motivate individual innovative behavior in the context of March’s exploration-exploitation tradeoff [4, 5].

There is extensive prior research on innovation and creativity. For example, researchers investigate the relationship between innovation and different types of leadership [6, 7], corporate culture [8], competition [9], personality traits [10–13], socio-demographic characteristics [14–16] or individuals’ motives [17].

One strand of literature focuses on how monetary incentives shape innovation and creativity, with somewhat mixed results. For example, while piece-rate pay leads to greater effort compared to a flat wage, it fails to increase creative output [18]. Further experimental evidence documents that large financial incentives decrease creativity [19], tournament incentives do not lead to higher creativity in open tasks [20], linear and tournament incentives have only a very limited effect on creativity [21], and piece-rate incentives do not increase the originality of ideas [22]. In contrast to that, some studies show that financial incentives do have a positive impact on creativity. Performance bonuses have been shown to increase creative output [23]. Providing employees with rewards for their ideas substantially increases the quality of those ideas [24]. Bonus incentives increase performance in a non-routine analytical task that requires exploratory activities [25].

Because innovation involves the exploration of untested approaches, many of which are likely to fail, innovation is an inherently risky endeavor [26]. This makes interventions that manipulate the underlying risk of innovation promising tools for motivating exploratory behavior. Theoretical arguments posit that principals who want to motivate innovative behavior ought to implement compensation schemes that tolerate failure [27, 28]. Indeed, the empirical literature documents the innovation-inducing effects of contracts that allow for early failure and reward late-stage performance [5]. Similarly, funding policies that tolerate early failure and reward long-term success motivate innovation in scientific research [29]. A positive relationship between innovation and compensation schemes which protect managers from termination is also documented [30]. Also, innovation is documented to be greater when the bankruptcy code is debtor-friendly [31]. Lastly, more stringent dismissal laws foster employees’ innovative efforts because it reduces the risk of dismissal in case of short-term failure [32].

The existing literature on motivating innovation is primarily concerned with studying financial incentives that manipulate the underlying risk of the innovative process. However, changing incentive schemes within a firm can be both costly and organizationally demanding. We contribute to the literature because we study whether innovative behavior can be fostered by manipulating the mere *perception of the risk* of innovation—without manipulating the inherent *risk* of innovation itself. Manipulating the *perception of risk* is a promising avenue thanks to the well documented association between individual risk attitudes? that is the willingness to take risks? and innovative behavior [33]. For example, almost three-quarters of innovators exit with no equity, and entrepreneurship is shown to yield negative expected utility for individuals with common degrees of risk aversion [34, 35]. Further, risk tolerant individuals are more likely to become entrepreneurs [36], and entrepreneurs perceive themselves as less risk averse than managers and employees [37].

Our behavioral intervention relies on theoretical and experimental literature on salience, suggesting that shifting attention away from, or towards a specific feature, can smoothly guide decision-making processes [38, 39]. Numerous studies highlight the effectiveness of framing in directing risk perceptions [40–43]. The efficacy of interventions targeting risk perceptions has been extensively investigated in health behavior [44–46] and in investment settings [47–49].

We bridge the gap between the literature on motivating innovation and on risk salience by asking the following research question: Can innovative behavior be (de)motivated by making the risk of innovation less (more) salient? Our intervention shall affect innovative behavior by guiding participants risk perceptions, while holding monetary incentives constant. Hence, we abstain from manipulating the inherent risk of innovation. To address our research question, we conduct an experiment in a controlled laboratory setting and consider innovation in the context of March’s exploration-exploitation tradeoff [4]. This tradeoff is characterized by the allocation of resources between the exploitation and fine-tuning of known strategies and the uncertain exploration of new opportunities. Innovative behavior is measured based as individual exploratory behavior in a business game.

Participants are randomly assigned to either a control group or one of two treatment groups. Treated participants are required to describe either (i) the business strategy they implemented, with the goal of diverting the attention away from the outcome of innovation by emphasizing the process of innovation, and hence, making the riskiness of exploration less salient, or, (ii) the outcome of the business strategy they implemented in terms of the achieved business profits, emphasizing the outcome of innovation with the goal to make the riskiness of exploratory activities more salient. Because of the risk of failure when pursuing exploration, focusing participants away from (towards to) this risk is hypothesized to make participants explore more (less) often.

**Hypothesis 1a** *Participants reporting on the implemented business strategy explore more than participants in the control group*.
**Hypothesis 1b** *Participants reporting on the outcome of the implemented business strategy explore less than participants in the control group*.

Subsequently, we investigate the mechanism that drives the hypothesized effect. We posit that participants’ attention is shifted to the salient aspect of the exploration-exploitation task: Participants reporting the business strategy should pay more attention to the strategic business choices while participants reporting the outcome of the implemented strategy should pay more attention to the profits achieved.

**Hypothesis 2a** *Participants reporting the implemented business strategy provide more attention to the strategy than the control group*.
**Hypothesis 2b** *Participants reporting on the outcome of the business strategy provide more attention to the profit than the control group*.

Lastly, we expect that the effect of risk salience on exploratory behavior works through the channel of risk attitudes. An intervention changing the perceived risk should affect risk-averse individuals more strongly than risk-neutral participants. The more risk-averse a participant, the more their innovative behavior should be inhibited by an increased perception of risk, or be promoted by a decreased perception of risk. We thus hypothesize heterogeneity in the treatment effect and suppose that our interventions specifically affect risk-averse participants. This line of reasoning aligns with the previous literature documenting that making the incentive structure more risk-tolerant leads to a behavioral change particularly among the risk-averse participants [5].

**Hypothesis 3a** *Risk-averse participants assigned to the strategy treatment explore more than risk-averse participants in the control group*.
**Hypothesis 3b** *Risk-averse participants assigned to the profit treatment explore less than risk-averse participants in the control group*.

The outlined hypotheses were pre-registered before data collection at the AEA RCT registry (ID: AEARCTR-0005214), which is The American Economic Association’s registry for randomized controlled trials. Sampling and data collection follow a sequential analysis procedure, and data analysis adheres to the pre-analysis plan. Both documents were also pre-registered before data collection.

## Materials and methods

### The lemonade stand task

Participants were tasked with managing a virtual lemonade stand, a paradigm developed and first employed to study innovation by [5]. In this business game, participants need to make decisions on multiple parameters, such as the lemonade’s recipe (i.e., the sugar content, the lemonade content, the color of the lemonade), the location of the lemonade stand and the price of a cup of lemonade. Those five choice variables can be combined in 23,522,994 different ways. See Fig 5 in S1 File for a screen shot of the decision screen.

The game lasts for 20 periods. The participants’ goal is to maximize the profit of the virtual lemonade stand, as they are compensated accordingly: Participants receive 50% of the profits of their lemonade stand that were generated during all 20 periods, representing a standard pay-for-performance scheme.

In the task, participants face uncertainty since they do not know the profits associated with a chosen business strategy. Each combination of the five choice variables leads to a different profit, which is unknown to the participants before trying the respective strategy. At the beginning of the game, participants receive a default strategy through a letter that was written by a fictitious previous manager. In this letter, the previous manager informs participants about the choice combination that she chose to implement and the profits that came along with that strategy. All participants receive the same default strategy, which is not the most profitable strategy. In period 1, the previous manager’s strategy appears as the default input on the screen by means of pre-populated fields. Participants can, of course, change the default field inputs to their preferred choice.

In each of the 20 periods, participants must decide on and implement a business strategy. After each period, participants are informed about the profit of the choices implemented. Additionally, they receive a brief customer feedback, which was an informative binary feedback: The computer randomly selects one of the three continuous choice variables (price, lemon or sugar content) and, then, provides an honest feedback on whether the value of the selected variable was too high or too low compared to the optimal value (e.g. “Your lemonade is too sweet”). Before the start of the experiment, participants received a pen, a blank sheet and a notes sheet (Fig 4 in S1 File), allowing them to take any notes and to keep track in each period of the implemented strategy and the associated profits in each period.

In this business game, participants face a tradeoff between *exploration* and *exploitation*. Exploitation denotes the fine-tuning of well-known approaches. Fine-tuning the previous manager’s strategy ensures secure and reasonable payoffs, but may prevent the discovery of superior approaches. Exploration instead comprises the implementation of new untested approaches—which can reveal superior approaches—but in the short term will likely also result in the discovery of inferior approaches and thus, failure [28]. Choosing to experiment with new unknown strategies, for example by changing the location of the lemonade stand and explore selling lemonade in a untested but potentially lucrative market, requires accepting the associated risk of failure.

The parameters are designed in such a way that over time, exploration will increase the chances of identifying the strategy that will lead to the global profit maximum, while exploitation leads to local profit maxima. The profit maximum in the default location, the business district, amounts to 100. The school is the most profitable location with a maximum profit of 200. In the stadium, the profit maximum amounts to 60. The profit maxima were unknown to the participants. Also, for each location there is a different optimal product mix. For example, the optimal sugar content is 1.5% in the business district, 9.5% in the school and 5.5% in the stadium. Note that all parameters in our experiment are exactly adapted from the original study [5]. A detailed description of the parametrization can be found in B.2. Sec in S1 File. The full experimental instructions are enclosed B.1. Sec in S1 File.

### Treatments

To test whether exploratory behavior can be altered by a salience intervention, we integrated a reporting stage into the game: In periods 3, 6, 9 and 12, participants were asked to submit a report. These reports shall induce an attention shift by making a specific aspect of the game relatively more salient, and consequently, another aspect of the game less salient.

We used a between-subject design and participants were randomly assigned to one of three groups. In the control group participants were not asked to report, constituting the benchmark. Participants in the profit treatment were asked to state the profits of their business. Participants in the strategy treatment were asked to describe the implemented strategy, i.e. the five chosen decision variables. The timing and description of the required reports was communicated by the instructions before the start of the game.

Note that the incentive structure between the groups was fully identical. The reports were not relevant for participants’ compensation, and reports were not shared during the game with neither another participant nor with the experimenter. This was all known to the participants. Hence, the treatment groups varied only in the content of the reports, but not regarding monetary incentives.

#### Control group

Participants assigned to the control group were not asked to submit a report.

#### Profit treatment

After periods 3, 6, 9, and 12, participants were asked to report the profits of their lemonade stand realized in the last three periods. Along with the wording *“Please report your profits of the last three periods”*., participants faced an entry mask, whereby they needed to enter the profits of each of the last three periods.

#### Strategy treatment

After periods 3, 6, 9, and 12, participants were asked to report the strategy they realized in the last three periods, i.e. the choice variables they implemented. Along with the wording *“Please describe your strategy in the last three periods. Why did you choose this strategy?”*, they faced a free form text field.

### Risk preferences

After participants finished the lemonade stand task, we elicited participants’ risk preferences in order to test hypotheses 3a and 3b. We employed the method developed in the Global Preference Survey [50]. This method is an experimentally validated survey procedure [51].

The procedure elicits participants’ risk preference based on a staircase lottery task and a self-reported willingness to take risks. In the lottery task, participants face a choice between either accepting a lottery or a safe option. Participants make five interdependent choices that result in the assignment of participants into one of sixteen categories. The self-reported variable asks participants to indicate their willingness to take risks on a 10-point Likert scale. Both variables are then z-score standardized and compounded to a single variable with experimentally validated weights, with the lottery tasks weighted by.47 and the self-reported variable by.53. We refer to the original paper for further details [51].

### Procedures

The study was approved by the IRB of the University of Fribourg under protocol number 471. Consent was obtained in written form before the beginning of the study.

We programmed the experiment in oTree [52]. The code is publicly available (see https://github.com/christianzihlmann/). We collected the experimental data in January 2020 at the laboratory of the University of Strasbourg (Laboratoire d’Économie Expérimentale de Strasbourg LEES). The subject pool at LEES consists mainly of students of the University of Strasbourg. Accordingly, participants in our sample are relatively young with a median age of 21, and well educated. Regarding gender, 51% of the participants identify themselves as females, 49% as males and no one indicated to be of diverse or other gender. See Table 4 in S1 File for further information on the sample characteristics.

Each of the four sessions lasted approximately 60 minutes. We used experimental currency units called Thalers with an exchange rate of 1:100 to €. All participants in the laboratory received a flat show-up fee of 2€, and in addition a performance-based variable payoff. Overall, the median as well as the average payoff was approx. 15€.

Participants were randomly assigned to the treatment and control groups, constituting the exogenous variation in this study. The random assignment was performed within-session in order to mitigate potential session-specific effects.

### Sampling

Before data collection, we elaborated a sequential analyses plan, following a method widely used in medicine and recently proposed for research in social sciences [53]. Sequential analyses allow for the running of studies with high statistical power in situations in which the effect size is difficult to accurately predict. Sequential analyses is efficient and the Type-1 error rate is controlled for [53].

We pre-determined an expected effect size and the smallest effect size of interest and strictly adhered to the procedure outlined in the sequential analysis plan. The sequential analysis plan was pre-registered before data collection (ID: AEARCTR-0005214).

The sequential analysis plan is based on the key outcome variable: the profit participants realized in the final period. In the final period, profit-maximizing participants should stick to the most profitable strategy that they discovered during all previous periods. Hence, the profit in the final period constitutes a suitable proxy for exploratory behavior in this experiment, adhering to the original study that designed and introduced the lemonade stand task [5].

In Ederer and Manso’s study [5], the effect size amounts to Cohen?s *d* = .725. We estimated our effects as lower, since our treatment interventions are based on a behavioral, non-monetary intervention rather than a change in the financial incentive scheme. As a best-estimate, we discounted the treatment effect by 30% and estimated an expected effect size of Cohen?s *d* = .5. The smallest detectable effect size of interest (SESOI) was set to *d* = .3875. Based on an expected effect size of Cohen?s *d* = .5, a power analysis indicated that for a two-sided test with an alpha of.05, a desired statistical power of.8, and two looks using a linear spending function, a total of 180 participants is needed (60 per group).

Three scenarios can arise on how to proceed after a first look at the data in the interim analysis (after 90 participants were collected which occurs at time *t* = .5). First, the data collection will be terminated if the observed effect size is smaller than the SESOI. Second, if the treatment effect is significant with an alpha boundary of.025, the data collection will also be terminated. Third, data collection will be continued if the interim analysis reveals i) an effect size larger than *d* = .5, but while *p* >.025 ii) the effect size to be between the smallest effect size of interest and the expected effect size. For controlling type 1 error rates, we use a linear spending function (power family function) [54]. For a more comprehensive description of the sampling procedure, see A Sec in S1 File.

As our main effect sizes are lower than the pre-determined SESOI (*d* <.3875), the first scenario came into force and we terminated data collection after collecting 90 observations, adhering to the procedure outlined in the sequential analysis plan. Therefore, the final sample consists of 90 participants, i.e. 30 participants in each group.

## Results

All data underlying the findings described in our manuscript are fully available without restriction at the following public repository address: https://osf.io/hup9c/.

As described in the pre-analysis plan and the previous section, we focus on i) the profit realized in the final period and ii) the maximum profit over all periods as proxies for exploratory behavior. Other proxy variables are analyzed in S1 File. The results remain qualitatively unchanged.

### Exploratory behavior

**Fig 1:**
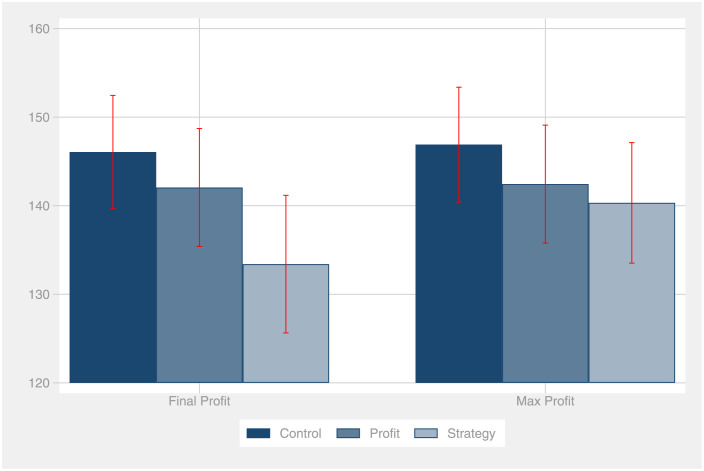
Means of the final and the maximum profits. The figure reports the means of the final and the maximum profits for each experimental group. Error bars indicate standard errors of the mean.

Fig 1 compares the means of the final and the maximum profits between the treatments. In contrast to our hypothesis 1a, participants in the strategy treatment do not explore more than the control group.

**Result 1a** *If anything, participants in the strategy treatment will explore less than participants in the control group*.
**Result 1b** *If anything, participants in the profit treatment will explore less than participants in the control group*.

On average, the control group realizes a final period profit of 146 thalers, compared to the strategy treatment with only 133 thalers. The same holds true for the maximum profit realized over the 20 periods. Thus, participants who were required to report their strategy explored less than the control group. The effect size amounts to *d* = .32, which is lower than the smallest effect size of interest. For this effect to be statistically significant with *p* ≤.05 and a power of 80%, we would need to collect 151 observations each, in both the control group and the strategy treatment. A two-sided t-test reveals a *p* value of *p* = .21. With the caveat of low statistical power in mind, the data suggests that our intervention backfired: Against our hypothesis, reporting the strategy does not increase, but decrease exploration.

In line with our hypothesis 1b, we find weak suggestive evidence that reporting the profits actually decreases exploratory behavior. Participants in the profit treatment realized a final profit of 142 thalers, which is four thalers less than the control group (146 thalers). However, the effect size is small (*d* = .11) and again lower than the SESOI. For this effect to be statistically significant with *p* ≤.05, we would need to collect 1254 observations each, in both the control group and the profit treatment. Accordingly, the effect is statistically not significant (*p* = .67) and indistinguishable from zero.

**Fig 2:**
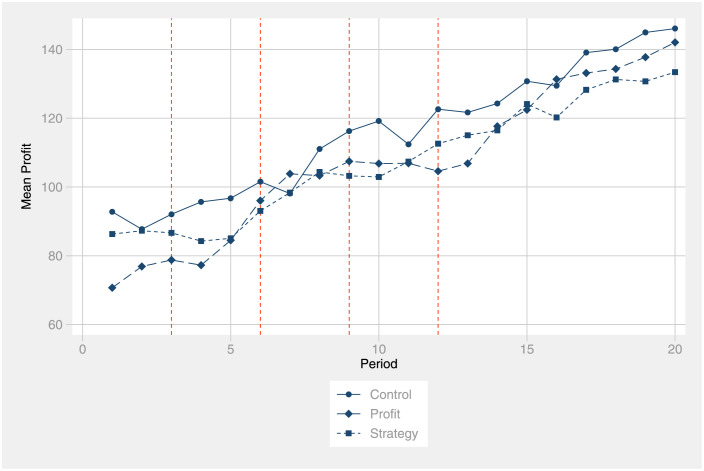
Evolution of profits over time. The figure reports the average profit by the experimental group for each of the 20 periods.

Fig 2 shows the evolution of the mean profit over the 20 periods for all three treatment groups. The red vertical lines represent periods that precede a reporting screen for the two treatment groups. Note that the average profit in the control group is higher than in the reporting treatments in nearly all periods and, crucially, already before participants needed to report the first time, which happened after period 3.

Therefore, as early as in the very first period, the control group seems to outperform the two treatment groups. Participants in the profit treatment earn significantly less profit in Period 1 (*p* <.01) than the control group. For the strategy treatment, the difference does not reach statistical significance. This pattern suggests that participants’ behavior in the treatment groups have already been affected by the announcement of the intervention. We will discuss the implications of this data pattern in the Discussion section.

### Note-taking behavior

Hypothesis 2 posits that the reporting interventions affect behavior because they shift participants’ attention. To measure attention, we construct a proxy measure that is based on exerted effort, which relies on data from a hard-copy notes sheet that participants could fill out voluntarily while managing the virtual lemonade stand, following [5]. Participants had the opportunity to write down their realized profits and/or the implemented strategic choices for each period in order to keep track of their record. We compute the proportion of filled out fields for each participant for the strategic choice variables and the periodic profit.

**Result 2a** *Participants in the strategy treatment do not pay more attention to the strategy than participants in the control group*.
**Result 2b** *Participants in the profit treatment pay significantly more attention to the profit than the control group*.

**Fig 3:**
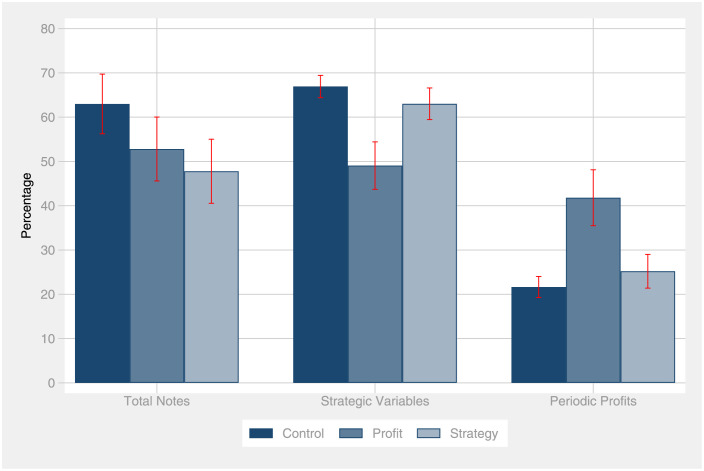
Note-taking behavior. For each experimental group, the figure reports the percentage of total notes taken as well as the means of participants? notes taken, in regards to the strategic decision variables, the periodic profits and the obtained feedback (relative to the total notes taken). Error bars indicate standard errors of the mean.

Fig 3 compares the percentage of notes taken. It illustrates what aspect held participants mainly focus on during the game.

Contrary to our hypothesis 2a, participants in the strategy treatment do not pay more attention to the strategic variables compared to the control group. While in the control, 67% of all fields related to the strategic choices have been filled out, this percentage is 63% in the strategy treatment. Statistically, this effect is indistinguishable from zero (*p* = .38, *d* = .25).

In line with our hypothesis 2b, we find that participants who were required to report their profits actually show a shift towards this dimension. The average participant in the profit treatment filled out 42% of the fields related to the periodic profit column, while the control group only filled out 22% of the fields. This effect is substantial and statistically highly significant (*p* <.01, *d* = .81). This finding is in line with our hypothesis: Through the reporting mechanism, we successfully shifted the attention of participants in the profit treatment towards the profit—and away from the business strategy. Participants in the profit treatment filled out 49% of the fields related to the strategic choice variables, which is significantly less than the control group with 67% (*p* <.01, *d* = .82).

We also collected the total time a participant spent in front of the decision screen. The time elapsed to make a decision on which business strategy to implement is another potentially valid proxy for attention devoted to the strategic choice variables. Indeed, the time in front of the decision screen highly significantly correlates with the notes taken on the strategic variables (*r* = .42, *p* <.01). This is why we find a similar pattern as in the analysis of notes-taking behavior. Contrary to our hypothesis 2a, participants in the strategy treatment do not devote more time to make up their strategic business choices (*p* = .37, *d* = .23). If anything, they spent less effort on the strategic choices than the control group, which is in line with the previously analyzed notes-taking behavior depicted in Fig 3. Note that this analysis of the response time was not pre-registered.

Taken together, while we find evidence in favor of hypothesis 2b, we need to reject hypothesis 2a.

### Risk-aversion and exploratory behavior

To test whether more risk-averse participants react stronger to the treatment induction, we regress exploratory behavior on the treatment dummies, the degree of risk aversion and interact those two regressors. Table 1 displays the results of OLS estimations.

**Table 1:** Heterogeneous treatment effect. OLS regression with robust standard errors (HC3) in parentheses. The outcome variable is the final profit (column (1)) and the maximal profit (column (2)), both representing exploratory behavior. Risk Aversion is the subject-specific degree of risk aversion. Level of significance: * p<.1; ** p<.05; *** p<.01.

	*Final Profit*	*Maximal Profit*
	(1)	(2)
Profit	-2.967	-3.290
	(9.99)	(10.01)
Strategy	-13.075	-7.592
	(10.73)	(10.09)
Risk Aversion	10.775	11.666
	(9.04)	(9.16)
Profit × Risk Aversion	-6.979	-7.961
	(11.38)	(11.45)
Strategy × Risk Aversion	-22.613	-28.083**
	(14.46)	(13.37)
Constant	144.634***	145.356***
	(7.10)	(7.14)
N	90	90
F	1.10	1.15
R^2^	.06	.07

The signs of the interaction terms are in line with our hypothesis. With an increasing degree of risk aversion, the negative treatment effects become stronger. This suggests that, as hypothesized, especially rather risk-averse participants react to the reporting mechanism that ought to alter the risk salience of the lemonade stand task. However, note that those heterogeneous treatment effects are statistically indifferent to the zero in model (1). In model (2), only the interaction term between strategy and risk aversion is statistically significant. The F-test of overall significance is low in both models and not significant, meaning that the model does not provide a better fit than the intercept-only model. Due to our limited sample size, results from investigating heterogeneous treatment effects, as presented in Table 1, should be taken with an appropriate portion of prudence.

**Result 3** *The data suggests that risk-averse participants react stronger to the interventions than risk-tolerant participants, but evidence is weak*.

## Discussion

### Discussion of the results

Our experimental findings are partly in line with the pre-registered hypotheses. As expected, participants in the profit treatment seem to explore less than participants in the control group. In contrast to our hypotheses, participants in the strategy treatment do not explore more but—if anything—*less* than participants in the control group. In this subsection, we elaborate and discuss potential explanations for this unexpected result.

#### Reputational concerns

Suppose participants feel reputational concerns when they need to report the strategies multiple times. Such thoughts may arise internally (from themselves) or externally (from the experimenter). The literature hypothesizes that reputational concerns lead to conservative behavior, i.e. less innovative and more risk-averse decisions [55–58]. As a result, participants may experience disutility of acting inconsistently: They do not change their strategy too often but consistently apply slight variations of the initial strategy, which results in less exploration.

Yet, participants would forgo potential profits when adhering consistently to a strategy in order to maintain their reputation. While we cannot fully rule out reputational concerns as a potential explanation for our findings, we note that if it was the driving force, participants in our experiment would take on substantial costs for maintaining their reputation.

#### Limited attention

Suppose participants have a limited attention span and can only exert a certain fixed level of effort. Participants in the strategy treatment then need to trade off the effort of writing the reports, with effort that they would otherwise invest in other activities such as the decision-making process—the latter being likely the more productive activity. Indeed, recent literature documents that people exhibit a limited span of effort [59]. Limited attention seems to be a reasonable argument, since requiring participants to report their strategy (or profits) is clearly an effort. In our data, we find patterns both for and against limited attention as the driving force.

On the one hand, participants in the strategy treatment do not only take fewer notes (Fig 3), but also spend less time on their decision-making process, as well as on analyzing the performance of the strategy implemented in the previous period (Fig 11 in S1 File). The final profit, representing exploratory behavior, positively and significantly correlates with all measures proxying effort (Total notes taken: *r* = .32, *p* <.01; Notes on strategic variables: *r* = .3, *p* <.01; Notes on periodic profits: *r* = .32, *p* <.01; Time spent on the decision screen: *r* = .22, *p* = .04; Time spent on the results screen: *r* = .27, *p* <.01.) Spending time on taking notes, or processing information on the decision and results screens, are productive activities as they can improve future decisions and thereby increase profits. The positive correlation between (productive) effort and exploration suggests that participants in our strategy treatment might have simply exerted less effort for productive tasks, resulting in a hampered performance.

On the other hand, we observe the change in innovative behavior as soon as the very first period, which speaks somewhat against limited attention as an appropriate explanation. In period 1 to 3, participants were not required to describe their strategy respectively the profits obtained and therefore did not need to trade off productive activities with unproductive effort for reporting. Nevertheless, we observe that participants who needed to report their business strategy in round 3 perform worse than the control group already in the first round of the business game.

While we believe that limited attention explains some of the unexpected negative effect of the strategy treatment, the data patterns are inconclusive and allow for other potential channels.

#### Backfiring intervention: Shifting attention *toward* risk

The underlying behavioral mechanism might be that our strategy intervention backfired because we failed to shift attention away from risk but inadvertently shifted attention *towards* risk. In the profit treatment, we arguably made the profit and with it, the risky aspect of the game, particularly salient. The data confirms this hypothesis. Consequently, as hypothesized, participants seem to explore less.

In the strategy treatment, we intended to shift attention to the strategic variables, making risk less salient. However, the strategic choice variables may accompany and be inseparable from the profit. Thus, requiring participants to report their strategy may have simultaneously focused them on the profit as well. Because participants in the strategy treatment reported their choices, the risk of losing money may have been more salient than in the control group. Indeed, when text analyzing the reports that participants in the strategy treatment submitted after period 3, we find that 40% of the participants (12 out of 30) explicitly mention, and make a reference to, the profit—even though participants were only explicitly asked to report on the implemented strategies. This suggests that we might have unintentionally lead participants in the strategy treatment to focus on the risky aspect of the business game.

If risk becomes more salient, the exploratory behavior likely decreases and performance may be inhibited. This channel would match with the observation that the treatment effect already occurs from the very beginning. The mere awareness of needing to report may have been enough to increase the salience of risk.

Clearly, with the available data at hand we are unable to corroboratory pin down the exact behavioral mechanism at work, which leads our intervention to backfire. This discussion is therefore not conclusive, but shall rather give an overview of potential and likely channels.

### Limitations and avenues for future research

Our study enhances the knowledge of existing research on innovation. We augment the evidence regarding tools that foster innovation by studying a non-monetary salience intervention and its effects on innovative behavior. While previous research primarily focused on whether financial incentives can increase innovation, we design a mechanism with the goal of altering participants’ perception of risk. This opens up interesting paths for future research because behavioral interventions might be promising and cost-effective approaches to help organizations boost employees’ innovative behavior. We also add to the literature on changing risk perceptions. While behavioral interventions have been shown to be able to influence risk perceptions, this has not yet been extensively tested in the context of innovative behavior. Lastly, we believe this study also constitutes a methodological contribution by adhering to rigorous approaches such as pre-registration, sequential analyses and the reporting of inconclusive results.

However, the present study also suffers from limitations that call for future research. First, our experiment investigates the effect of salient interventions on exploratory behavior in the context of March’s exploration-exploitation tradeoff [4]. The findings thus do not necessarily generalize to creativity, which is not considered an element of this tradeoff. However, both creativity and exploration share common aspects, and are both characterized by the risk of failure. For a more granular perspective on the difference between innovation and creativity, see [7]. Future research could address whether creativity can be fostered through a salience mechanism.

Second, our findings do not generalize to entrepreneurial behavior. We do not measure variables that are associated with entrepreneurship, nor do we survey participants about entrepreneurial activities or behavior. Future research could fill this gap.

Third, measuring attention is a difficult challenge. In addition to choice data, we collected process data and measured the number of notes taken as well as response times. Both of these variables arguably proxy attention, however, imperfectly so. Future studies could mitigate this issue by including more sophisticated process tracing techniques such as eye-tracking.

Fourth, our study design does not feature a principal-agent relationship. Future research could implement such a relationship and investigate the effect on agents’ innovative behavior in a situation in which the principal actively implements a monitoring or control mechanism, and relate those findings to the literature on potential adverse effects of control [60]. Moreover, given that innovation is inherently associated with a risk of failure, the nature of the principal-agent relationship—for example the extent of mutual trust—may be crucially important in the context of shaping exploratory behavior.

Lastly, we adhered to our pre-determined procedure and stopped the data collection after the interim analysis, because the effects for both treatments groups were lower than the smallest effect size of interest defined in the sequential analysis plan. Consequently, our sample size is not large enough to make reliable inferences given the low effect sizes we observe. Therefore, our results should be interpreted prudently.

## Conclusion

Identifying and evaluating measures that effectively foster innovative behavior is highly relevant for leaders and decision-makers in organizations [3]. We experimentally investigate whether salience can serve as a tool to promote exploratory behavior. Indeed, we find that making the risk of innovation more or less salient may affect innovative behavior. However, our behavioral intervention aiming to foster innovation turns out to be ineffective and to even jeopardize exploratory behavior. We discuss potential reasons for why the intervention backfired.

Our results may have important implications for practitioners and researchers alike. First, we show that purely behavioral interventions that do not change monetary incentives likely do affect innovative behavior. Second, even interventions that are carefully derived from the existing literature can turn out to be ineffective and may even backfire.

Hence, interventions need to be tailored with diligence and require meticulous evaluation before implementation, ideally through experimental methods [61, 62]. This is especially important since interventions have become increasingly popular due to their simplicity and alleged effectiveness [63–65]. However, RCTs conducted in so-called nudge units show substantially lower effect sizes than RCTs in published academic studies [66]. The authors show that publication bias can account for the full difference. Also, replicated studies are documented to have on average a 33% lower effect size than the original studies [67]. By adhering to rigorous methods such as pre-registration and the publication of papers unconditional of the results, effect sizes will better reflect reality compared to the past [68].

## Supporting information

S1 File Appendix. S1 File contains the Appendix of the article, including sections related to the sequential analyses procedure, the experimental design, the elicitation of control variables and further results.
(PDF)

S2 File Pre-analysis plan. S2 File contains the pre-analysis plan that was pre-registered before data collection.
(PDF)
