# Supplementary material for: Fostering innovation: Experimental evidence on the effectiveness of behavioral interventions
Source: PLoS One. 2022 Oct 19;17(10):e0276463. doi: 10.1371/journal.pone.0276463 (PMC9581361; doi:10.1371/journal.pone.0276463)
Supplement: S1 File — contains the Appendix of the article, including sections related to the sequential analyses procedure, the experimental design, the elicitation of control variables and further results. (PDF) [file pone.0276463.s001.pdf]

Supporting Information S1 File

A Sequential Analysis

Our sequential analysis plan followed the summarized procedure outlined below. It concerned the key outcome variable, the profit in the final round. The assessment on how to proceed after the first stage was based on a hypothesis test conducted with a two sample t-test.

Based on an expected effect size of Cohen's $d = 0.5$, a power analysis indicated that for a two-sided test with an alpha of 0.05, a desired statistical power of 0.8, and two looks using a linear spending function, a total of 180 participants is needed (60 per group). If the expected difference is significant at the first interim analysis (after 90 participants or time = 0.5, with an alpha boundary of 0.025) the data collection will be terminated. The data collection will also be terminated if the observed effect size is smaller than the smallest effect size of interest, which is set at $d = 0.3875$ based on the researcher's willingness to collect at most 300 participants for this study, and the fact that with one interim analysis, 300 participants provide 0.8 power to detect an effect of $d = 0.3875$. If the interim analysis reveals an effect size larger than 0.5, but while $p > 0.025$, the data collection will be continued until 60 participants per group have been collected. If the effect size lies between the smallest effect size of interest ($d = 0.3875$) and the expected effect size ($d = 0.5$), the planned sample size will be increased based on a conditional power analysis to achieve a power of 0.9 (or to a maximum of 100 participants per group, or 300 participants in total). The second analysis is performed at an alpha boundary of 0.0358.

In the original paper of [5], participants in the pay-for-performance contract yield on average a profit in the last period of 111 thalers. Because our control group is mimics one-to-one the pay-for-performance group in [5], we assume that our control group will yield 111 thalers in the last period too. Furthermore, based on a small test conducted in September 2019, we expect a standard deviation of $SD = 40$. It is our best assumption that the standard deviation is equal for all three groups.

Next, we define the smallest detectable effect size of interest. Based on practical limitations,

namely budget restrictions, we are willing to collect at most 300 observations in total. To identify an effect with 80% power when comparing the control with one of the two treatment groups, with one interim look at the data, the effect must be at least 15.5 thalers large, translating in a Cohen's $d = 0.3875$. We deem such an effect also from a real-life perspective as appropriate - implementing a new reporting policy comes along with costs, and thus, the beneficial or detrimental effect should be large enough to be of practical relevance.

Next, we elaborate on the expected effect. In [5], the exploration contract yields in the final period on average a profit of 140. This leads to a profit difference between pay-for-performance and exploration contract of $29(= 140 - 111)$. With a $SD = 40$, this yields a Cohen's $d = 0.725$. Most probably, our effects will be lower since our treatment interventions are based on a behavioral mechanism but do not change monetary incentives, as this is the case in [5]. As a best-estimate, we expect our effect to be 30% (or 9 thalers) lower than in their study. Therefore, we estimate that participants in the strategy treatment will yield a lower profit in the last period than their exploration contract, and we estimate this to be at 131. This yields an effect size of $20(= 131 - 111)$, or a Cohen's $d = 0.50$. For the profit treatment, we cannot base our estimates on a previous study due the lack of comparable alternatives. However, we expect the profit treatment to perform similarly as the strategy treatment (but in the opposite direction, of course). Consequently, we adopt the same Cohen's $d = 0.5$ for the profit treatment. Based on the expected effect size of $d = 0.5$, with power 0.8 and an alpha of 0.05, we obtain a sample size $n = 60$ per group or $n = 180$ in total (after both looks). We will have a first look at time $= 0.5$, that is when 90 participants are collected.

For controlling type 1 error rates, we use a linear spending function (power family function), as outlined in [54]. The alpha of 0.05 for a single look is adjusted for sequential analyses, namely for two looks using a linear spending function, yielding a nominal alpha of 0.0586. Thus, planning on analyzing the data at two different stages of the experiment, that is with one interim analysis, we formulate the analysis plan outlined in the main body of the text.

B Experimental Design

B.1 Instructions

Welcome

You are now taking part in a scientific study. Please read the following instructions carefully. Everything that you need to know in order to participate in this experiment is explained below. Should you have any difficulties in understanding these instructions, please notify us. We will answer your questions at your cubicle. During the course of the experiment you can earn money. The amount that you earn during the experiment depends on your decisions. All the gains that you make during the course of the experiment will be exchanged into cash at the end of the experiment.

The exchange rate will be: 100 thaler = 1 EUR

The experiment is divided into 20 periods. In each period you have to make decisions, which you will enter on a computer screen. The decisions you make and the amount of money you earn will not be made known to the other participants - only you will know them. At the end of the experiment, you will be requested to respond to survey questions. Please note that communication between participants is strictly prohibited during the study. Communication between participants and unnecessary interference with computers will lead to the exclusion from the study. In case you have any questions don't hesitate to ask us.

Procedures

In this experiment, you will take on the role of an individual running a lemonade stand. There will be 20 periods in which you will have to make decisions on how to run the business. These decisions will involve the location of the stand, the sugar and lemon content and the lemonade color and price. The decisions you make in one period, will be the default choices for the next period. At the end of each period, you will learn what profits you made during that period. You will also hear some customer reactions that may help you with your choices in the following periods.

Letter from the Previous Manager

The previous manager of the lemonade stand has left you guidelines on how to run the business.

The letter from the previous manager is the following:

Dear X,

I have enclosed the following guidelines that you may find helpful in running your lemonade stand. These guidelines are based on my previous experience running this stand. When running my business, I followed these basic guidelines:

Location: Business District

Sugar Content: 5.2%

Lemon Content: 7.0%

Lemonade Color: Green

Price: 8.2 thaler

With these choices, I was able to make an average profit of about 85 thaler per period. I have experimented with alternative choices of sugar and lemon content, as well as lemonade color and price. The above choices were the ones I found to be the best. I have not experimented with alternative choices of location though. They may require very different strategies.

Regards, Previous Manager

Note that in the first period, these choices will appear as defaults.

Compensation

Your compensation will be based on the profits you make with your lemonade stand. You will get paid 50% of your total lemonade stand profits during the 20 periods of the experiment.

For example, if your total profits during the 20 periods of the experiment were 1700 thaler, you will earn 850 thaler, worth 8.50 EUR.

In addition, you will earn the show-up fee of 2 EUR.

Report

Displayed only if assigned to *profit treatment*: Please report your profits of the last three periods.

Displayed only if assigned to *strategy treatment*: Please describe your strategy in the last three periods. Why did you choose this strategy?

B.2 Parametrization, Notes Sheet and Screen Shot

We adopted the experimental parameters from [5]. The participants could make following choices:

- Location = {Business District, School, Stadium}
- Sugar Content = {0,0.1,...,20}
- Lemon Content = {0,0.1,...,20}
- Lemonade Color = {Green, Pink}
- Price = {0,0.1,...,10}

The optimal product mix in each location is shown in table 2.

For the profit calculation in each location, a linear penalty function was implemented. So if the participant did not implement the optimal choices, she was penalized according to the values summarized in table 3. In each location, the penalty factors represented in the table are associated with a deviation of one unit for each of the variables. Note that we implemented a minimum of 0, i.e. participants could not earn negative profit.

Table 2. Optimal Product Mix, by Location

	Business District	School	Stadium
Sugar	1.5%	9.5%	5.5%
Lemon	7.5%	1.5%	5.5%
Lemonade Color	Green	Pink	Green
Price	7.5	2.5	7.5
Maximum Profit	100	200	60

Table 3. Penalty factors, by Location

	Business District	School	Stadium
Sugar	3	6	0.5
Lemon	3	6	0.5
Lemonade Color	20	60	0.5
Price	3	6	0.5

Fig 4. Notes sheet. The sheet was distributed to participants with a pen at the start of the experiment.

Note Table

To help you keep track of your choices and outcomes in each period, we have included the following table. Please use this table if you want to take any notes during the course of the experiment.

**Period**	Location	Sugar Content	Lemon Content	Lemonade Color	Price	Profit	Feedback
**1**							
**2**							
**3**							
**4**							
**5**							
**6**							
**7**							
**8**							
**9**							
**10**							
**11**							
**12**							
**13**							
**14**							
**15**							
**16**							
**17**							
**18**							
**19**							
**20**							

Fig 5. The following screen shot provides an illustration of the decision screen that participants faced, here round 1.

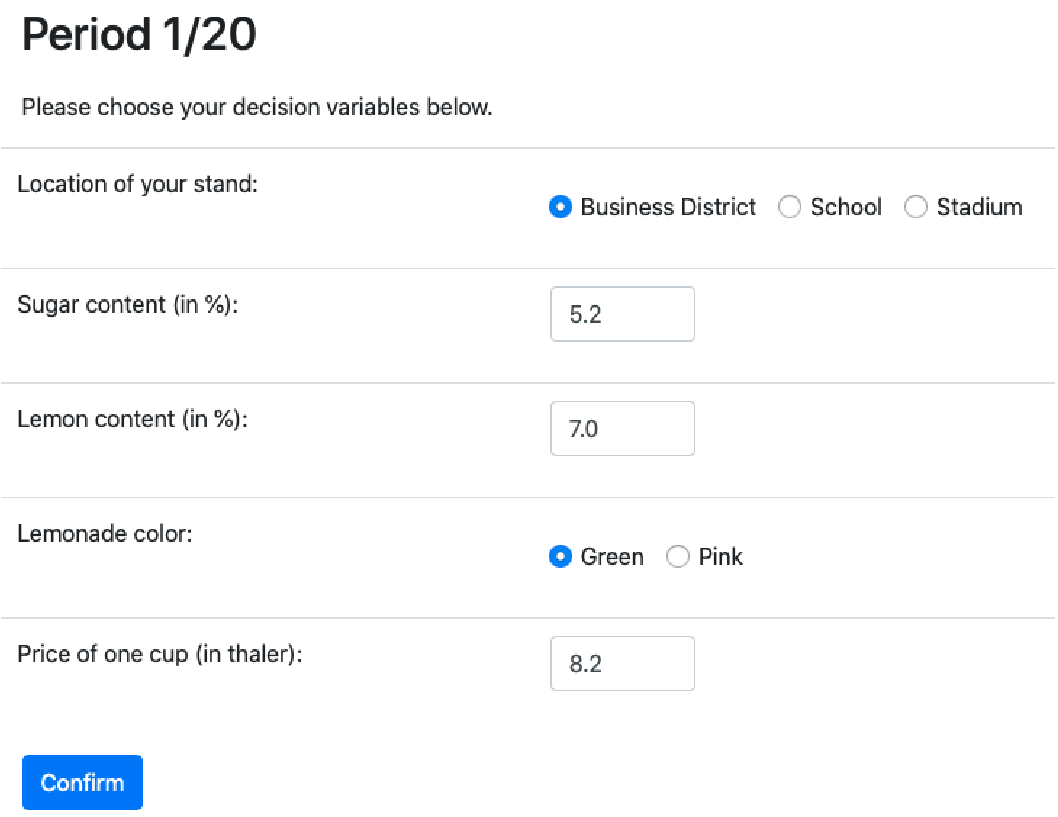

C Elicitation of Control Variables

C.1 Demographics

What is your gender?

What is your age?

What is the highest level of education you have completed or the highest degree you have received?

What is your academic field?

What is your nationality? (If more than one apply, select the one you feel is most representative for you).

In what country did you grow up? (If you grew up in more than one country, please indicate the country you lived the longest while growing up).

In what country do you currently reside?

Do you identify yourself with any of the following religions? ['Christianity', 'Judaism', 'Islam', 'Buddhism', 'Hinduism', 'Other', 'No religion / Atheism']

D Results

D.1 Demographics

Table 4. Demographics

		Freq.	Percent	Cum.
Age				
	18	14	15.56	15.56
	19	10	11.11	26.67
	20	11	12.22	38.89
	21	18	20.00	58.89
	22	15	16.67	75.56
	23	7	7.78	83.33
	24	5	5.56	88.89
	25	4	4.44	93.33
	26	2	2.22	95.56
	31	1	1.11	96.67
	34	2	2.22	98.89
	37	1	1.11	100.00
	Total	90	100.00	
Gender				
	Male	44	48.89	48.89
	Female	46	51.11	100.00
	Total	90	100.00	
Education (completed)				
	High school	39	43.33	43.33
	Bachelor or equivalent	30	33.33	76.67
	Master or equivalent	21	23.33	100.00
	Total	90	100.00	

D.2 Performance and Exploratory Behavior

We investigate different proxies for exploratory behavior, in line with [5]. Table 5 provides an overview of all measured outcome variables.

Some of these variables reflect choices or are constructed based on choices by the participants and proxy their explorative behavior: the variables based on the location choice indicate whether the subject detected the profit-maximizing location. This is impossible when exclusively following the customer feedback. Further, the higher the standard deviation for the

Table 5. Overview of proxies for exploratory behavior

Variable	Description
final_profit	Profit in final round, continuous variable (min:0,max: 199.1).
max_profit	Highest profit in all rounds, continuous variable (min:0,max: 199.1).
overall_profit	Sum of total profit of all 20 periods, continuous variable (min:0,max: 3982).
final_location	Final location chosen, categorical variable (School, Business, Stadium).
location_non-default	Constructed variable. Count of chosen non-default locations, i.e. non-Business locations. Discrete variable (min:0, max: 20)
max_exploration_phase	Constructed variable. Longest duration of an exploration phase. An exploration phase starts when participants choose a location other than the default location suggested by the previous manager. An explorative phase is defined as ending when a subject switches back to the default location or when a subject does not change location and lemonade color and also does not change lemon content, sugar content and price by more than 0.25 units. Discrete variable (min:0, max: 20). Adapted 1:1 from EM.
duration_exploration_phase	Constructed variable. Total duration of all exploration phases. Discrete variable (min:0, max: 20)
std_dev_sugar	Constructed variable. Standard deviation for sugar choices over all rounds. Continuous variable.
std_dev_lemon	Constructed variable. Standard deviation for lemon choices over all rounds. Continuous variable.
std_dev_price	Constructed variable. Standard deviation for price choices over all rounds. Continuous variable.
average_std_dev	Constructed variable. The average subject-specific standard deviation of strategy choices for the three continuous variables sugar, price, lemon. Continuous variable.

continuous variables (sugar, lemon, price) is, the more explorative the subject behaved. Instead, the outcome variables with respect to profits mirror how these choices are translated into payoffs. Since the business game is designed such that explorative behavior increases the chance of finding a profit-increasing strategy, those outcome variables should be closely correlated. This can be seen by Table 6.

Table 6. Spearman Cross-correlation table of exploration outcome measures

Variables	finalprofit	maxprofit	overallprofit	final_loc_binary	loc_non-def	expl_phase_maxdur	expl_phase_totdur	sd_sugar_1-10	sd_sugar_11-20	sd_lemon_1-10	sd_lemon_11-20	sd_price_1-10	sd_price_11-20	sd_choices_1-10	sd_choices_11-20
finalprofit	1.000														
maxprofit	0.9759	1.000													
	0.000														
overallprofit	0.9057	0.8849	1.000												
	0.000	0.000													
final_loc_binary	0.7661	0.7385	0.7379	1.000											
	0.000	0.000	0.000												
loc_non-def	0.7414	0.7398	0.7976	0.7417	1.000										
	0.000	0.000	0.000	0.000											
expl_phase_maxdur	0.5948	0.6265	0.561	0.53	0.6373	1.000									
	0.000	0.000	0.000	0.000	0.000										
expl_phase_totdur	0.7105	0.7365	0.6668	0.6621	0.78	0.8447	1.000								
	0.000	0.000	0.000	0.000	0.000	0.000									
sd_sugar_1-10	0.3501	0.354	0.3469	0.2708	0.2526	0.42	0.3211	1.000							
	0.0007	0.0006	0.0008	0.0098	0.0163	0.000	0.002								
sd_sugar_11-20	0.1565	0.1908	0.0678	0.1731	0.0898	0.2411	0.2858	0.0744	1.000						
	0.1407	0.0716	0.5255	0.1027	0.4001	0.022	0.0063	0.486							
sd_lemon_1-10	0.3827	0.382	0.3232	0.282	0.1787	0.2807	0.1973	0.4654	0.1184	1.000					
	0.0002	0.0002	0.0019	0.0071	0.0919	0.0074	0.0623	0.000	0.2664						
sd_lemon_11-20	0.3859	0.4099	0.2915	0.3927	0.2923	0.3651	0.4654	0.2855	0.4309	0.2593	1.000				
	0.0002	0.0001	0.0053	0.0001	0.0052	0.0004	0.000	0.0064	0.000	0.0136					
sd_price_1-10	0.3692	0.404	0.2295	0.3056	0.2304	0.3379	0.3536	0.1681	0.2825	0.2455	0.3087	1.000			
	0.0003	0.0001	0.0296	0.0034	0.0289	0.0011	0.0006	0.1133	0.007	0.0197	0.0031				
sd_price_11-20	0.3748	0.4211	0.1951	0.2727	0.1418	0.286	0.3913	0.1525	0.3483	0.2084	0.3267	0.4113	1.000		
	0.0003	0.000	0.0653	0.0093	0.1825	0.0063	0.0001	0.1514	0.0008	0.0487	0.0017	0.0001			
sd_choices_1-10	0.3228	0.3509	0.334	0.1311	0.1557	0.2363	0.2124	0.4962	0.1365	0.4954	0.2001	0.2161	0.1471	1.000	
	0.0019	0.0007	0.0013	0.2183	0.1429	0.0249	0.0444	0.000	0.1997	0.000	0.0586	0.0408	0.1665		
sd_choices_11-20	0.6152	0.6263	0.6248	0.2486	0.392	0.3907	0.442	0.4483	0.0882	0.4197	0.3367	0.1404	0.1712	0.7068	1.000
	0.000	0.000	0.000	0.0182	0.0001	0.0001	0.000	0.000	0.4083	0.000	0.0012	0.187	0.1067	0.000	

Results with respect to the final profit and the maximum profit were already discussed in the main body of the article. In line with these discussed results, the mean overall profit in the control group is higher than in both reporting treatments, as shown by Fig 6.

Fig 6. Mean of overall profit by treatments

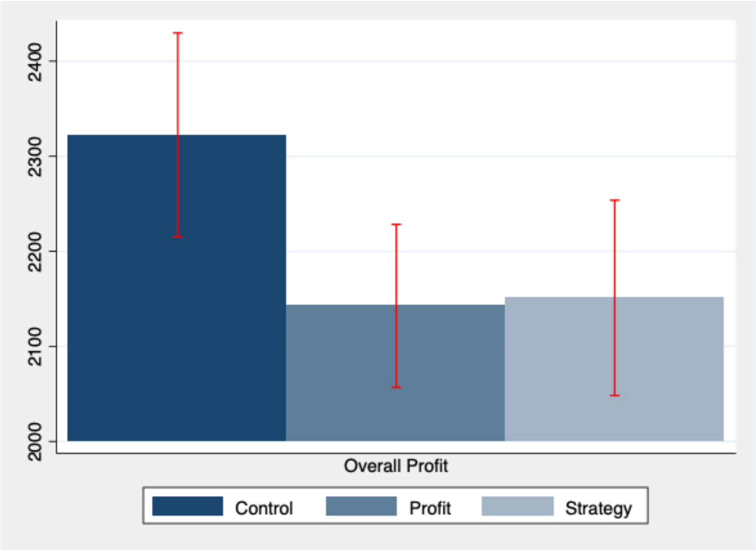

Analyzing the location chosen in the final round of the game shows the same pattern. There are three possible locations, with the school being the optimal one. As Fig 7a depicts, around 80% of participants in the control group chose the optimal location in the final round. This proportion is higher than in the profit (appr. 73%) and strategy treatment (appr. 66%). These difference-in-means are not statistically significant (two-sided Mann-Whitney U-tests: p=0.5449 for control group vs. profit treatment, p=0.2469 for control group vs. strategy treatment).

Results with respect to the exploration phases further support our finding, see Fig 8: the control group explores more than the profit and the strategy treatment. However, these differences are, again, not significant.

Lastly, Fig 9 shows that also the results for all outcome variables with respect to the standard deviations are in line with the previous results: the standard deviation in the control group is slightly higher than in the reporting treatments. As expected, the choices within the first ten rounds of the business game vary more than the choices in the last ten rounds. This

Fig 7. Location measures

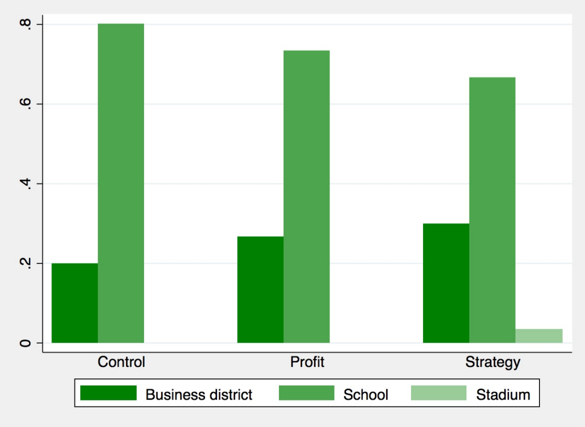

(a) Proportion of participants by Location in final round.

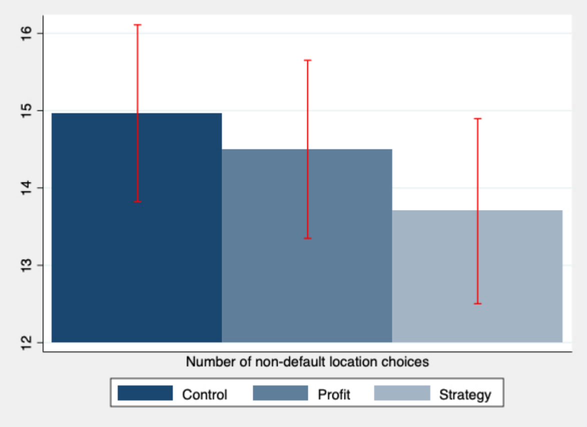

(b) Number of times the non-default location was chosen.

Fig 8. Exploration phase measures

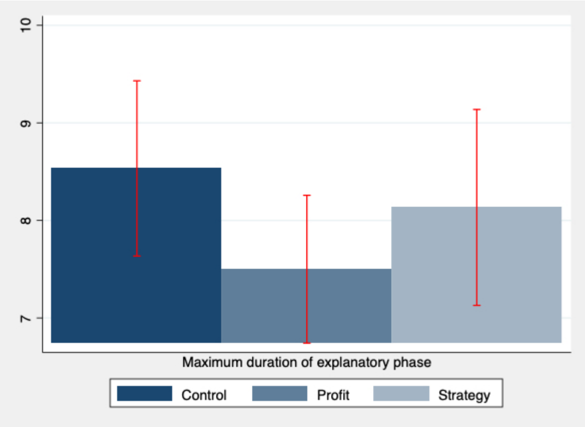

(a) Maximum of all exploration phases

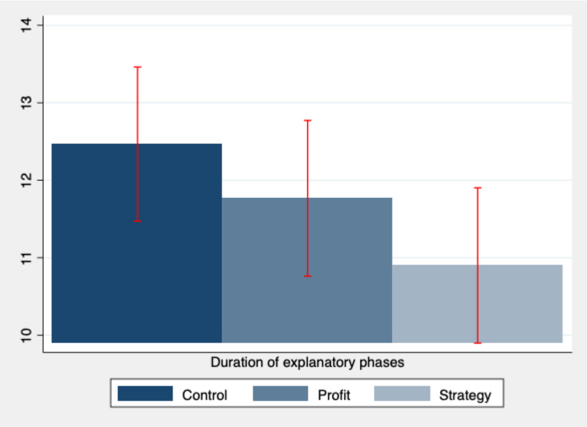

(b) Duration of all exploration phases

Notes: The figure reports the means of the maximum length and of the duration of all exploration phases . Error bars indicate standard errors of the mean.

early exploration is intuitive since the individual can profit from her findings for a longer time horizon than later stage exploratory activities.

Fig 9. Standard deviation measures

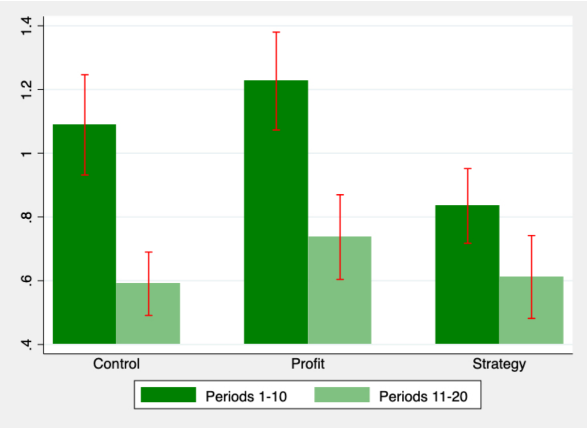

(a) Standard deviation of sugar choices over all rounds.

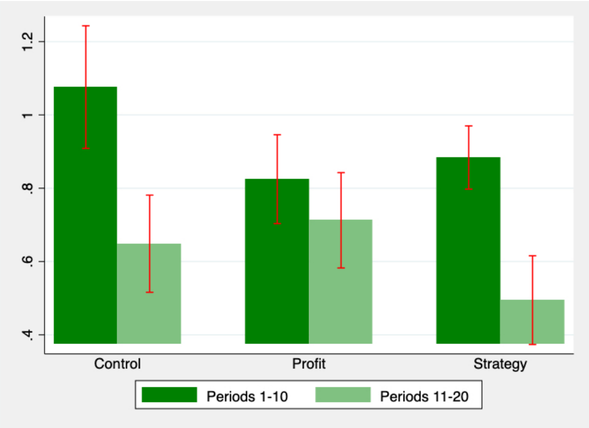

(b) Standard deviation of lemon choices over all rounds.

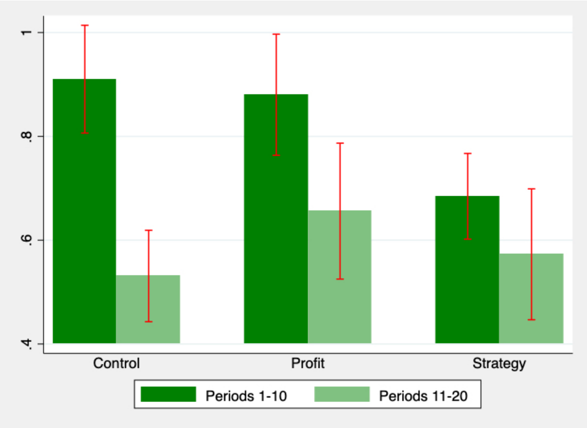

(c) Standard deviation of price choices over all rounds.

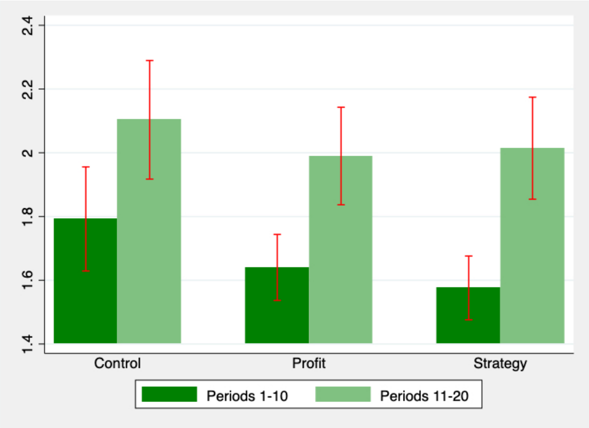

(d) Standard deviation of all choices over all rounds.

To further analyze the explorative behavior of our participants, we compare the average subject-specific standard deviation of the profits. The variability of profits in all treatments is higher in periods 1-10 (Fig 10). However, the standard deviation of the profits is not different between the treatments. Two-sided Mann-Whitney U-tests: p=0.8941 for periods 1-10 and p=1.000 for periods 11-20 between control group and profit treatment. p=0.5444 for periods 1-10 and p=0.1833 for periods 11-20 between control group and strategy treatment and p=0.4598 for periods 1-10 and p=0.3142 for periods 11-20 between profit and strategy

treatment.

Fig 10. Standard deviation of realized profits.

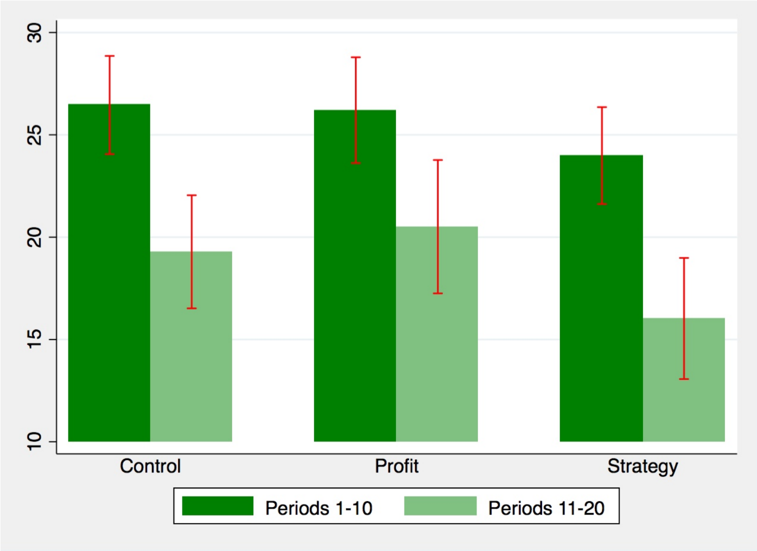

D.3 Exerted effort and attention

We analyze different proxies for exerted effort, again, in line with [5]. Table 7 provides an overview of all measured proxies. Table 8 regress the various proxies for effort on the experimental group dummy, a gender dummy and the degree of risk aversion.

Table 7. Overview of proxies for effort

Variable	Description
total_time_decision	Time elapsed for all 20 Decision-Screens, continuous variable.
focus_time_decision	Focus time elapsed for all 20 Decision-Screens, continuous variable.
total_time_result	Time elapsed for all 20 Result-Screens, continuous variable.
focus_time_result	Focus time elapsed for all 20 Result-Screens, continuous variable.
total_time_reporting	Time elapsed for all 20 Reporting-Screens. Treatment groups only, continuous variable.
focus_time_reporting	Focus time elapsed for all 20 Reporting-Screens. Treatment groups only, continuous variable.
total_notes	Proportion of filled out fields in notes sheet, continuous variable.
notes_strategic	Proportion of notes with respect to strategic variables relative to total_notes, continuous variable.
notes_profit	Proportion of notes with respect to profits relative to total_notes, continuous variable.
notes_feedback	Proportion of notes with respect to customer feedback relative to total_notes, continuous variable.

Fig 11. Effort measured by time elapsed

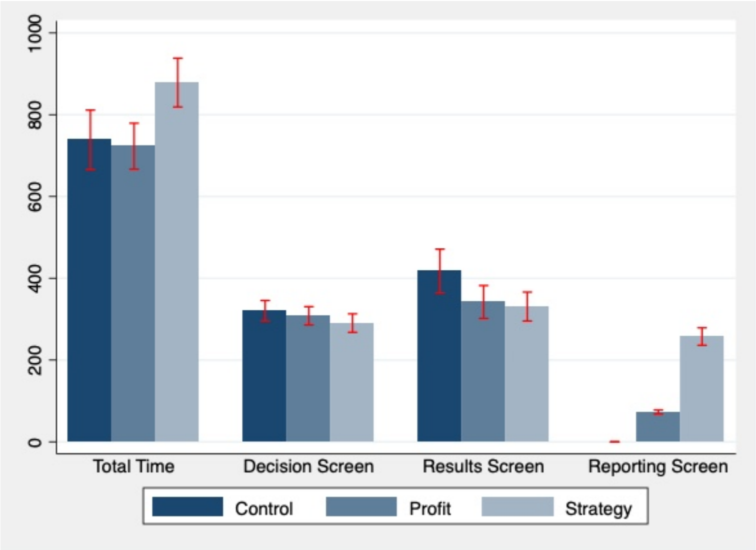

Notes: The figure reports the means of the participants time elapsed for the total time, the time spent at the decision screen, at the results & feedback screen, and at the reporting screen, respectively.

Table 8. Regressions

	Dependent variables:					
	Overall Time	*Decision Time*	*Results Time*	*Strategy Notes*	*Profit Notes*	*Feedback Notes*
	(1)	(2)	(3)	(4)	(5)	(6)
Profit Treatment	-17.982	-12.933	-77.952	-0.147	0.068	-0.103
	(90.19)	(33.36)	(62.17)	(0.11)	(0.09)	(0.11)
Strategy Treatment	144.199	-27.883	-80.104	-0.167	-0.123	-0.083
	(92.52)	(34.22)	(63.78)	(0.11)	(0.09)	(0.12)
Gender	33.602	-4.345	21.356	0.167*	0.204***	0.104
	(75.42)	(27.90)	(51.99)	(0.09)	(0.08)	(0.09)
Riskaversion	33.238	9.500	39.170	0.092	0.094	0.027
	(77.35)	(28.61)	(53.32)	(0.09)	(0.08)	(0.10)
Constant	704.299***	317.549***	385.747***	0.486***	0.642***	0.471***
	(81.23)	(30.05)	(55.99)	(0.10)	(0.08)	(0.10)
N	90	90	90	90	90	90
R^2	0.046	0.011	0.036	0.092	0.152	0.027
Adjusted R^2	0.001	-0.035	-0.009	0.049	0.112	-0.019

Level of significance: *p<0.1; **p<0.05; ***p<0.01
