# Supplementary material for: Fostering innovation: Experimental evidence on the effectiveness of behavioral interventions
Source: PLoS One. 2022 Oct 19;17(10):e0276463. doi: 10.1371/journal.pone.0276463 (PMC9581361; doi:10.1371/journal.pone.0276463)
Supplement: S2 File — contains the pre-analysis plan that was pre-registered before data collection. (PDF) [file pone.0276463.s002.pdf]

Supporting Information S2 File

Pre-Analysis Plan

Nudging innovation: the effect of salience

Elisa Matthewes[†], Anis Nassar[‡] and Christian Zihlmann[†]

January 19, 2020

Abstract

Innovation is a core component of a growing economy and firm survival. Yet, it is still to be fully understood how to best foster it. Contrary to existing studies discussing the effect of incentive schemes on innovative behavior, we focus on non-monetary interventions and experimentally investigate whether shifting attention to the strategy or to the profit affects the innovative behavior of participants in a business game. Subjects manage a virtual lemonade stand facing a trade-off between exploring and exploiting a given strategy in each period. To nudge subjects' innovative behavior, we introduce a reporting stage and exogenously vary the salience of either their profit or their strategy throughout different rounds of the business game: While subjects in the Profit Treatment are regularly asked to describe their latest profits, subjects in the Strategy Treatment describe which strategy they followed within the preceding rounds. In a Control Group, subjects are not reporting anything. We expect that participants who are asked to report their strategies will be more innovative than those who report their profits. Results of our study could have implications for organizational decision-makers aiming to promote innovative employee behavior.

† *Chair of Industrial Economics, University of Fribourg, Switzerland*
‡ *Chair of Microeconomics, University of Fribourg, Switzerland*

1 Introduction

Innovation is at the core of technological progress, it drives economic growth and is thus of utmost importance (Schumpeter (1942)). It is required in many contemporary jobs (Shalley et al. (2000), Unsworth (2001)) and is a driver of firm performance and even survival (Nystrom (1990)). Stimulating innovation is hence one of the highest concerns of CEOs (Rudis (2004)). The literature investigating effects of leadership or personal traits on innovation is consequently abundant. Yet, little is known about the organizational devices firms can use to unleash the innovative potential of their employees.

A natural tool to direct employees' efforts is the financial incentive scheme. For example, fixed wages and pay-for-performance (P4P) where employees are rewarded if they meet or exceed certain performance measures, are widely used schemes. The main motivation to use P4P over fixed wages is that, because the pay reflects performance, agents under P4P increase their effort, which in turn increases their productivity. This effect is established and has been well documented on individuals and teams in empirical research as well as lab and field experiments in a diverse set of tasks such as retailing, windshield installing, fruit picking or repeatedly typing a paragraph (Lazear (2000), Bandiera et al. (2007), Dickinson (1999), Friebel et al. (2017)). However, these studies have in common that tasks are simple, effort-based, and do not allow for innovation. On the other hand, recent research shows that when agents have to perform a task where they can innovate –and innovating potentially improves or worsens their performance– agents do increase their effort but at the same time decrease their innovative behavior (Ederer & Manso (2013), Azoulay et al. (2011)). It appears that P4P has, in this context, conflicting effects. First, a standard motivating effect: as the agent has a stake in his performance, he increases his effort. Second, an innovation inhibiting effect: the more risk averse the agent is, the more reluctant he is to innovate and potentially to lose some of his payoff, if the pursued innovation fails. Agents under fixed wage perform no better as they exert low effort and low innovation given they have no stake in the outcome. This suggests standard payoff schemes are not satisfactory for innovative tasks as either effort or innovative behavior is inhibited.

Consequently, a recent strand of literature investigates how novel payoff schemes can be designed to best foster innovative behavior. In a lab experiment, Ederer & Manso (2013) (EM) conclude that an "exploration contract" that allows for early failure while rewarding late stage performance can overcome the innovation inhibiting effect of P4P, while keeping its effort inducing quality. But many companies today still use P4P and changing these incentive schemes can both be costly and organizationally demanding. We thus investigate a softer intervention while keeping the P4P scheme fixed. We introduce an attention shift, or salience shift, aimed

at overcoming its innovation inhibiting aspect. To do so, we replicate the experimental design of EM, where participants run a virtual lemonade stand over 20 periods and try to maximize its profits, and exogenously vary the salience of either the profit or the strategy by introducing reporting and varying the report's content.

The use of P4P schemes by firms implies periodic reporting to evaluate the agent's performance. It is then natural to introduce reporting in our experiment, that we will utilize as the tool to generate the shifts in salience. We therefore ask participants to produce reports and vary the content of said reports to be either outcome focused –describing profits– or process focused –describing strategies–. By doing so, we investigate how the contextual focus of the reporting interacts with the innovative behavior of participants under an effort inducing P4P scheme. As a control, we also replicate exactly the conditions of EM in their P4P treatment and thus also implement a *no reporting* treatment. In the *profits reporting* treatment, the profits generated in the stand are assuredly the salient part of the task. They are both the determinant of the participant's final payoffs as well as the sole content of the reporting. This treatment group is expected to have the lowest levels of innovation. The expected dynamics inhibiting agents' innovation under the *profits reporting* is that the more risk averse the participant, the less likely she is to try uncertain innovative strategies. In the *strategies reporting* treatment however, the attention of the participant is shifted to her strategy by making it the sole content of the reporting. The participants in this treatment group are expected to be the most innovative and have the highest profits, with a larger effect for the more risk averse.

We expect our results to have implications for organizational decision-makers, namely those who work with P4P schemes. Indeed, if it is shown to be fruitful, introducing strategy reporting or changing existing reporting to a more strategy focused one, is relatively easy to implement. Our proposal to foster the innovative potential of employees thus has the advantage to be easily feasible, while still rewarding performance at every stage. To illustrate, think of a sales employee. We would advise to reward him based on his sales performance, but ask him to report what he does to reach his goals. In a nutshell: *Reward performance, but ask about strategies.*

2 Literature

First, a semantic clarification about innovation. Innovation and creativity are complex and intertwined concepts. To avoid confusion on which we are addressing, we adhere to the definition of Hughes et al. (2018) to delineate the two. In this experiment, we will investigate innovation, not creativity. Exploring different strategies for our participants does not include

an idea generation stage or the generation of something absolutely novel, which are characteristics of the creative process. It does however include idea implementation, adopting and adapting others' idea and the *product* of their successful innovation is a functioning and implemented strategy, not an idea. These are characteristics of the innovation process. However, we also review literature regarding creativity given, as noted by other authors such as Anderson et al. (2004), part of the research so far has covered the two with overlap.

Dating back at least to the Roman and Greek empires (Greene (2000)), or more recently to Schumpeter (1942), the quest to better understand innovation has captivated entrepreneurs, economists and firms alike. Still today, an abundant literature is produced to understand which channels impact it and how to better foster it. Recent research has focused on different effects such as personal characteristics, leadership, financial incentives, or processes (e.g. reporting). These different strands of research can be split in, on one side, investigating the individual determinants of innovation and on the other, the organizational ones. While interesting effects have been uncovered in this study we do not focus on the personal characteristics but on organizational enablers or inhibitors to innovation. Still, given their potential interaction with our study, we control for personal traits such as risk aversion and *Big 5*.

The incentivisation of tasks, to it's most common form in P4P, is long documented to be an effective organizational tool to increase productivity. Lazear (2000) shows that the productivity of windshield installers in Safelite Glass Corporation increased when management changed their compensation from fixed wages to piece-rate pay. Shearer (2004) finds similar evidence in a randomized field experiment with Canadian tree planters. Dickinson (1999) shows that subjects in a laboratory experiment type more letters when their compensation is more sensitive to performance. However, these findings are applicable to effort-based tasks whereas P4P can actually undermine performance when tasks require creativity or innovation as have noted psychologists since decades (e.g. McGraw (1978), Kohn (1993)) and more recently economists (e.g. Amabile (1996), Ederer & Manso (2013)). While this may be true, EM show that it is not an imperative. They argue that P4P inhibits innovation mainly through the channel of risk aversion, as agents are less likely to innovate if they perceive they can be worse off by following uncertain paths. Subtle variations in the incentivisation, namely allowing for an incentive free, and so risk free period, or for a *golden parachute* if the innovation does not pay off, can decrease the perception of said risk and releases the innovative potential of participants. We thus follow a similar path and aim to decrease the perception of risk of the participants, although without changing the incentive scheme from a P4P.

Because agents are limited in the attention that they can allocate to information when making decisions (DellaVigna (2009)), making certain information more or less salient can influence

the decision making process. Köszegi & Szeidl (2013) and Bordalo et al. (2013) both show that in multi-attributes decision making, agents overweight certain attributes simply because they stand out, or are more salient, and not necessarily because they are the most important. Importantly, Bordalo et al. (2012) show that the over-weighting of salient attributes also applies to decisions under uncertainty leading decisions makers to context-dependent representations of risk. By making the strategy more salient, we expect participants to shift their attention away from profits and thus from the risk and be more attentive to their possibility to innovate. We anticipate this will at least partly alleviate the innovation-inhibiting effect of P4P. Our contribution is thus twofold: First, we test the effect of salience with regards to innovation. Second, we test the effect of salience by making not a specific information more salient, but a process.

3 Research Strategy

3.1 Procedures and Subject Pool

To address our research question, we will collect and analyze experimental data at the laboratory of the University of Strasbourg (Laboratoire d'Économie Expérimentale de Strasbourg LEES). The experiment will be programmed and conducted with oTree (Chen et al. (2016)). Participants will be recruited from the LEES subject pool using an online recruitment system. The experiment will be conducted in French.

One session will last approximately 60 minutes. We will use experimental currency units called thalers with an exchange rate of 1:100. Subjects receive EUR 2 show-up fee. We expect the average profit (in addition to the show-up fee) to amount between EUR 14 to EUR 18.

Subjects will be randomly assigned to the treatment and control groups, constituting the exogenous variation in this study. The random assignment is performed within-session in order to mitigate potential session-specific effects. For the determination of sample size and power calculations, refer to 3.4.

3.2 Experimental Design

Our experimental task is adapted from Ederer & Manso (2013). Subjects solve a task in which they are facing a trade-off between exploration and exploitation: Participants manage a virtual lemonade stand. Over 20 experimental periods, participants decide on multiple parameters such as the recipe of the lemonade (sugar and lemonade content as well as color), the location

of the lemonade stand and the price of a cup of lemonade. The possible combinations of these choice variables amounts to 23'522'994 combinations[1]. Participants are compensated according to the realized profits. Thus, participants aim is to maximize the profit of the lemonade stand, and with it, their own earnings. Participants do not know the profits associated with each of the available choices. Participants receive a default strategy, i.e. the choices and the associated profit of a fictitious previous manager. Naturally, the default strategy is not the most profitable strategy.

After each period, participants learn the profit for the implemented choices. Also, they receive a brief customer feedback, implemented by having the computer randomly select one of the three continuous choice variables (price, lemon or sugar content) to provide a binary feedback. Consequently, the feedback is only informative for the location in which the subject chose to sell in the current period.

The task is characterized by an exploration-exploitation trade-off with two main behaviors: either fine-tuning the default strategy and yielding a profit similar to the previous manager (exploitation), or experimenting with new strategies and taking the associated risk of failure but also the chance of success (exploration). Parameters are designed in way that exploration will increase chances to identify the strategy that leads to the global maximum while exploitation rather leads to local maxima. The parameters to calculate the profits of the lemonade stand are one-to-one adapted from EM.

The payoff is determined by a standard pay-for-performance incentive scheme: participants are paid 50% of the profits they make during all the 20 periods.

3.2.1 Treatments

We implement a reporting mechanism into the game: In periods 3,6,9 and 12, subjects are requested to report. We exogenously and randomly vary the focus of reporting, inducing an attention shift through making a specific aspect of the game salient. Three different groups are implemented, one receiving no treatment, and two receiving the actual treatments outlined below[2].

1. Control
   No reporting. Mimics 1-to-1 EM pay-for-performance treatment.
2. Profit treatment
   After the decisions are made, in period 3,6,9 and 12, subjects are requested to report

[1](3) *(2(19*10+1*9)) * (2) * (9*10+1*9)
[2] Note that treatments do not impact incentives.

their profit of the last three periods. Along with the wording *"Please report the profit of the last three periods."*[3], subjects will face an entry mask, where they need to enter the profits of the last three periods.

3. Strategy treatment
   After the decisions are made, in periods 3,6,9 and 12, subjects are requested to report their strategy of the last three periods. Wording: *"Please describe your strategy in the last three periods. Why did you choose this strategy?"*.[4]

The treatments shall induce an attention shift through making a specific aspect of the game more salient, i.e. the process (strategy) or the outcome (profit). By manipulating the salient part of the task, we expect to nudge the participants judgement and decisions.

3.2.2 Elicitation of individual characteristics

After subjects completed the experiment, we elicit the following individual characteristics through a survey: demographics, risk preferences (Falk et al. 2018) and Big-5 (Lang et al. 2011).

3.3 Hypotheses

We are interested in how a salience shift affects exploratory activities of our agents. Subjects assigned to the *strategy treatment* shift their attention to the strategy. Building on theoretical predictions of the salience literature (e.g. Bordalo et al. (2013)) and on findings by Ederer & Manso (2013), we consequentially expect subjects in the *Strategy treatment* to explore more than in our *Control Group*[5]. Subjects in the *Profit treatment*, however, shift their attention to the profit, but not the determinants of it. In line with salience literature, we expect subjects in the *profit treatment* to explore even less than subjects in the *Control Group*.

Behavioral Prediction 1a: For subjects assigned to the *strategy treatment*, the salient aspect of the task is the strategy. They will shift their attention to the strategy (the process) and will therefore explore more than without intervention.
Hypothesis 1a: Subjects in the *strategy treatment* will exhibit a higher exploration behavior than subjects in the *Control group*.

[3] In French: "Reportez vos profits des trois périodes précédentes."

[4] In French: "Décrivez votre stratégie(s) durant les trois précédentes périodes. Pourquoi avez-vous choisi cette(s) stratégie(s)?"

[5] There are several outcome variables which represent exploration behavior. Refer to section 4 for a formal formulation of the hypotheses.

Behavioral Prediction 1b: For subjects assigned to the *Profit treatment*, the salient aspect of the task is the profit. They will shift their attention to the profit (the outcome) and will therefore explore less than without intervention.
Hypothesis 1b: Subjects in the *Profit treatment* will exhibit a lower exploration behavior than subjects in the *Control group.*

After formulating the main hypothesis that our treatment affects behavior and results in higher (lower) exploration behavior for the strategy (profit) treatment, we subsequently investigate the mechanism driving this effect. We posit that the mechanism driving Behavioral Prediction 1a and 1b is a shift of attention to the salient aspect (the strategy, respectively the profit).

Behavioral Prediction 2a: For subjects assigned to the *strategy treatment*, the salient aspect of the task is the strategy. They will shift their attention to the strategy (the process), while for the control, there is no attention shift due to the non-existing intervention.
Hypothesis 2a: Subjects in the *Strategy treatment* will provide more attention to the strategy than the *Control group.*

Behavioral Prediction 2b: For subjects assigned to the *Profit treatment*, the salient aspect of the task is the profit. They will shift their attention to the profit (the outcome) while for the control, there is no attention shift due to the non-existing intervention.
Hypothesis 2b: Subjects in the *Profit treatment* will provide more attention to the profit than the *Control group.*

We expect that the attention shift works through the channel of risk-aversion. Risk-aversion leads to conservative exploitation behavior in our experimental task. If risk-averse subjects are in the control group, the risk of loosing money (through the potential failure of the newly tested exploratory business strategy) is salient. In the *profit treatment*, the aspect of potentially loosing money becomes more salient since after three periods, subjects have to report their profits in each round. On the other hand, subjects in the *strategy treatment* are focused on the strategy. The risk of loosing money becomes less salient because the decision variables are the salient part of the game. Thus, to conclude, we expect a heterogeneous treatment effect: We hypothesize that our treatment manipulation works through shifting attention away from the inherent risk attached to the game.[6]

[6] Previous studies have revealed a heterogeneous performance in this task Ederer & Manso (2013), Herz et al. (2014). Thus, we expect that we will face different types in our population. We expect them to exhibit a very different exploration behavior. Hypotheses 3 relies on the assumption that risk-aversion identifies these groups. However, the degree of risk-aversion could fail to identify these groups. Hence, we will also fit a finite-mixture model in order to test our hypothesis that performance in this task is heterogeneous (and

Behavioral Prediction 3: Risk-averse subjects will react more to the treatment induction.
Hypothesis 3a: Risk-averse subjects assigned to the *profit treatment* will explore less than risk-averse subjects in the *Control group.*
Hypothesis 3b: Risk-averse subjects assigned to the *strategy treatment* will explore more than risk-averse subjects in the *Control group.*

3.4 Sampling

In order to efficiently perform a high-powered study, we pursue a sequential analyses plan, following the method outlined in Lakens (2014). Our sequential analysis plan will follow the summarized procedure outlined below.[7] It concerns the key outcome variable (the profit in the final round). The assessment on how to proceed after the first look will be based on a hypothesis test conducted with a two sample t-test. All details of the approach are handled in the Appendix B: Power Analysis.

Based on an expected effect size of Cohen's d = 0.50, a power analysis indicated that for a two-sided test with an alpha of .05, a desired statistical power of .8, and two looks using a linear spending function, a total of 180 participants are needed (60 per group). If the expected difference is significant at the first interim analysis (after 90 participants or time = .50, with an alpha boundary of .025) the data collection will be terminated. The data collection will also be terminated when the observed effect size is smaller than the smallest effect size of interest, which is set at d = 0.3875 based on the researcher's willingness to collect at most 300 participants for this study, and the fact that with one interim analysis 300 participants provide .8 power to detect an effect of d = 0.3875. If the interim analysis reveals an effect size larger than 0.5, but while $p>.025$, the data collection will be continued until 60 participants (per group) have been collected. If the effect size lies between the smallest effect size of interest (d = 0.3875) and the expected effect size (d = 0.5), the planned sample size will be increased based on a conditional power analysis to achieve a power of .9 (or to a maximum of 100 participants per group, or 300 participants in total). The second analysis is performed at an alpha boundary of .0358.

treatment may affect only a certain type of agent), but that the defining characteristic of agent's heterogeneity is unobserved. Following alternative Hypothesis can be formed if observed risk-aversion fails to identify this heterogeneity: There are two types in our population. The two sub-populations are expected to behave and perform very differently in this task in general but also with regard to the intervention. We expect two types, call them "low" and "high" performers. Low performers will react less to the treatment intervention (be it strategy or profit) than high performers because they are more steadfast (e.g., due to risk or loss aversion, or aversion to experimentation).

[7] The power analysis is adapted to the outcome variable of main interest, the profit subjects yield in the last experimental period, that is in game period 20.

4 Empirical Strategy

Each subject will decide 20 times (once for each of the 20 periods). Therefore, we will have a total of 20 decisions. For each subject, decisions are correlated over time and thus, each subject constitutes only one observation. In general, unless noted otherwise, the tests are based on averages of the relevant variables of the individual subject. Further, standard errors are clustered at the individual level. For regression analysis, we will make use of the panel data structure which provides more statistical power. In this section, we will elaborate on the methods to test our hypotheses. For the formal statement of the hypotheses, please refer to the Appendix.

4.1 Key variables

4.1.1 Main endogenous variables

We are interested in the exploration behavior as outcome. Thus, the main endogenous variables are all measuring exploration behavior in different different ways and aspects. The following variables all represent exploration behavior - except the last two, which measure the second outcome of interest, namely attention. The outcome variables for the exploration behavior measure the same construct. They should be all highly correlated and thus yield the similar results when performing hypothesis tests.[8]

4.1.2 Main explanatory variables

Key explanatory variable is the dummy indicating the treatment (D). Further, risk preferences as well as Big5 are expected to explain our outcomes. We also include demographics. A detailed summary of all variables, incl. its elicitation and computation, is enclosed in Appendix 4.7.3.

4.2 Testing Hypothesis 1

The main tests which we will report are standard two-sample locational tests, comparing the control and the strategy treatment, and the control and the profit treatment: a two-sided

[8]However, we define a key outcome variable of interest, which is the **profit in the final period**. We also base our sample size calculation on this key outcome variable.

Variable Description	Properties	To test hypothesis
Profit in the final round	continuous min:0 max: 199.1	H1,H3
Location in the final period	Categorical: School, Business, Stadium	H1,H3
Maximal Profit in all rounds	continuous, min:0 max: 199.1	H1,H3
Overall profit	continuous, min:0 max: 3982	H1,H3
Longest duration of an exploration phase	discrete, min:0 max: 20	H1,H3
Total duration of all exploration phases	discrete, min:0 max: 20	H1,H3
Count of chosen non-default locations	discrete, min:0 max: 20	H1,H3
The average subject-specific standard deviation of strategy choices for the three continuous variables sugar, price, lemon	cont.	H1,H3
Proportion of subjects no choosing default location in first 10 rounds	cont, min:0 max: 1	H1,H3
Variance of profits between periods	cont.	H1,H3
Proportion of filled-out fields in the profit column	cont, min:0 max: 1	H2
Proportion of filled-out fields in the strategy columns	cont, min:0 max: 1	H2

Table 1: Main outcome variables

t-test along with a two-sided Mann-Whitney U test. In more detail, we apply the following tests.

Parametric

The basic model takes the following form:

$$Y_i = f(D) \tag{1}$$

where Y_i is the key outcome of interest, i.e. the outcome variables that represent exploration behavior, as defined previously and also in the Appendix. D is a dummy variable indicating the treatment condition.

To test hypothesis 1a and 1b, we apply first of all a standard two-sample two-sided t-test - primarily on the key outcome variable, the profit in the final period. To test robustness, we also test the other already mentioned outcome variables, as outlined previously. We apply to these again a t-test, or when more appropriate, an adequate test for the underlying parametric model (e.g. poisson).

Further, when running regression analysis, which allows for inclusion of control variables, we employ the following linear regression model, making use of the panel structure of our dataset (with clustered standard errors on the individual level).

$$Y_{it} = \beta_0 + \beta_1 D_i + \epsilon_{it} \tag{2}$$

with

Y_{it} :=	endogenous variable representing a measure for exploration behavior for *individual i* in *period t*
D_i :=	dummy variable indicating if *individual i* is assigned to the respective treatment group (=1) or in the control (=0), representing the **exogenous condition**
ϵ_{it}:=	**random error term**, white noise, normally distributed

Some outcome variables, such as the location in the final period, are categorical. For those, we will estimate a probit and logit model where the dependent binary variable takes the value 1 if the final location choice is the school, and 0 otherwise. The independent variables are binary variables and indicate the treatment group.
Other outcome variables, such as the proportion of subjects not choosing the default location in the first 10 rounds, will also be tested with a probit and logit model, where the dependent binary variable takes the value 1 if the subject chose at least once another location than the default location in the first 10 periods, and 0 otherwise. The independent variables are binary

variables and indicate the treatment group.
We use also the semi-parametric Cox-hazard rate model. This allows to test if subjects continue to explore once they entered an exploratory phase[9].
Lastly, some outcome variables may be highly right-skewed due to their nature (count data), e.g. the count of chosen non-default locations. There, we will apply the appropriate parametric model (GLM: poisson, quasi-poisson or negative bi-nominal regression, zero-inflated if necessary). Table 4.2 gives an overview about the expected adequate parametric model for each of the outcome variable.

Non-Parametric

To test hypothesis 1 non-parametrically, we will employ Mann-Whitney-Wilcoxon ranksum tests (when averages apply: of the individual subject averages over the 20 periods[10]) between the control group and other treatment groups on the endogenous variables mentioned in section 4.1.1. As a robustness check, we will also bootstrap our sample (999 replications, seed 55) and run a t-test afterwards. Also, we will perform a non-parametric kernel regression.

4.3 Testing Hypothesis 2

To measure attention, we will construct a measure based on the effort sheet subjects fill out: We will compute the proportion of filled out fields for each subject, for the columns of the strategy choices as well as for for the profit columns.

Parametric

The basic model takes the same form as in Hypothesis 1. To test hypothesis 2, we apply a t-test on the average of the relevant variable of the individual subject. Further, we make use of the panel structure of our dataset and employ the same linear regression model as for Hypothesis 1 (OLS, panel regression model with clustered standard errors on individual level).

[9] Following EM, we classify subjects as having entered an exploratory phase as soon as they chose a location other than the default (business district). An explorative phase ends when i) a subject switches back to the default location (business district) or ii) when a subject does not change location and color and simultaneously does not change the three continuous variables (lemonade content, sugar content, price) by more than 0.25 units.

[10] We first test the averages for all 20 periods but in a next step, we do so for period 1-10 and period 11-20 separately because we expect subjects explore more in the first half.

Variable Description	Properties	Parametric model
Profit in the final period	continuous min:0 max: 199.1	OLS (robust regression)
Location in the final period	Categorical: School, Business, Stadium	Logit
Maximal Profit of all periods	continuous, min:0 max: 199.1	OLS (robust regression)
Overall profit	continuous, min:0 max: 3982	OLS (robust)
Longest duration of an exploration phase	discrete, min:0 max: 20	Poisson or quasi-poisson (or OLS)
Total duration of all exploration phases	discrete, min:0 max: 20	Poisson or quasi-poisson (or OLS)
Count of chosen non-default locations	discrete, min:0 max: 20	Poisson or quasi-poisson (or OLS)
The average subject-specific standard deviation of strategy choices for the three continuous variables sugar, price, lemon	cont.	OLS (robust)
Proportion of subjects no choosing default location in first 10 rounds	cont, min:0 max: 1	Probit
Variance of profits between periods	cont.	OLS (robust)
Proportion of filled-out fields in the profit column	cont, min:0 max: 1	OLS (robust)
Proportion of filled-out fields in the strategy columns	cont, min:0 max: 1	OLS (robust)

Table 2: Expected parametric model

Non-Parametric

To test hypothesis 2 non-parametrically, we will employ Mann-Whitney-Wilcoxon ranksum tests of the individual subject averages of filled out fields, over the 20 periods[11] between the control group and other treatment groups. As a robustness check, we will run a bootstrap t-test (999 replications, seed 55).

4.4 Testing Hypothesis 3

For hypotheses 3a, 3b and 3c, we split our sample of participants at the median level of risk-aversion. We then, in essence, compare the treatment groups to the control group separately for each type of risk-aversion (low or high).

Parametric

First of all, we apply two-sided two-sample t-tests to compare treatment groups and control group based on the type of risk aversion, namely low or high risk-aversion based on a median split. This is the main analysis.

Next, as a robustness check, we apply the continuous risk-aversion variable and we apply the following regression model:

$$Y_i = \beta_0 + \beta_1 D_i + \beta_2 RA_i + \beta_3 D_i \times RA_i + \epsilon_i \tag{3}$$

whereas:

$Y_i :=$ endogenous variable representing a measure for exploration behavior for *individual i*

$D_i :=$ dummy variable indicating if *individual i* is assigned to the respective treatment group (=1) or in the control (=0), representing the **exogenous condition**

$RA_i :=$ quasi-continuous variable, indicating the degree of risk aversion of each *individual i* (32 risk-aversion categories, standardized) representing the **degree of risk-aversion**

$\epsilon_i :=$ **random error term**, white noise, normally distributed

[11] We first test the averages for all 20 periods but in a next step, we do so for period 1-10 and period 11-20 separately because we expect subjects explore more in the first half.

Non-Parametric

First, we run Mann-Whitney U tests to compare risk-averse subjects assigned to the treatment and risk-averse subjects assigned to the control, and the same with risk-loving subjects (as outline above, based on a median split). As a robustness check, we will run bootstrapped t tests (999 replications, seed 55). Also, a non-parametric kernel regression serves as a robustness check.

References

Amabile, T. M. (1996), *Creativity in context*, Westview Press, Boulder, CO.

Anderson, N., De Dreu, C. K. & Nijstad, B. A. (2004), 'The routinization of innovation research: A constructively critical review of the state-of-the-science', *Journal of organizational Behavior* **25**(2), 147–173.

Azoulay, P., Graff Zivin, J. S. & Manso, G. (2011), 'Incentives and creativity: evidence from the academic life sciences', *The RAND Journal of Economics* **42**(3), 527–554.

Bandiera, O., Barankay, I. & Rasul, I. (2007), 'Incentives for managers and inequality among workers: Evidence from a firm-level experiment', *The Quarterly Journal of Economics* **122**(2), 729–773.

Bordalo, P., Gennaioli, N. & Shleifer, A. (2012), 'Salience theory of choice under risk', *The Quarterly journal of economics* **127**(3), 1243–1285.

Bordalo, P., Gennaioli, N. & Shleifer, A. (2013), 'Salience and consumer choice', *Journal of Political Economy* **121**(5), 803–843.

Chen, D. L., Schonger, M. & Wickens, C. (2016), 'otree—an open-source platform for laboratory, online, and field experiments', *Journal of Behavioral and Experimental Finance* **9**, 88–97.

DellaVigna, S. (2009), 'Psychology and economics: Evidence from the field', *Journal of Economic literature* **47**(2), 315–72.

Dickinson, D. L. (1999), 'An experimental examination of labor supply and work intensities', *Journal of Labor Economics* **17**(4), 638–670.

Ederer, F. & Manso, G. (2013), 'Is pay for performance detrimental to innovation?', *Management Science* **59**(7), 1496–1513.

Falk, A., Becker, A., Dohmen, T., Enke, B., Huffman, D. & Sunde, U. (2018), 'Global Evidence on Economic Preferences*', *The Quarterly Journal of Economics* **133**(4), 1645–1692.
URL: *https://doi.org/10.1093/qje/qjy013*

Friebel, G., Heinz, M., Krueger, M. & Zubanov, N. (2017), 'Team incentives and performance: Evidence from a retail chain', *American Economic Review* **107**(8), 2168–2203.

Greene, K. (2000), 'Technological innovation and economic progress in the ancient world: Mi finley re-considered', *Economic history review* pp. 29–59.

Herz, H., Schunk, D. & Zehnder, C. (2014), 'How do judgmental overconfidence and overoptimism shape innovative activity?', *Games and Economic Behavior* **83**, 1–23.

Hughes, D. J., Lee, A., Tian, A. W., Newman, A. & Legood, A. (2018), 'Leadership, creativity, and innovation: A critical review and practical recommendations', *The Leadership Quarterly* **29**(5), 549–569.

Jennison, C. & Turnbull, B. W. (2001), 'Group sequential tests with outcome-dependent treatment assignment', *Sequential Analysis* **20**(4), 209–234.
URL: *https://doi.org/10.1081/SQA-100107646*

Kohn, A. (1993), *Punished by Rewards: The Trouble with Gold Stars, Incentive Plans, A's, Praise and Other Bribes*, Houghton Mifflin, Boston.

Köszegi, B. & Szeidl, A. (2013), 'A model of focusing in economic choice', *The Quarterly Journal of Economics* **128**(1), 53–104.

Lakens, D. (2014), 'Performing high-powered studies efficiently with sequential analyses', *European Journal of Social Psychology* **44**(7), 701–710.
URL: *https://onlinelibrary.wiley.com/doi/abs/10.1002/ejsp.2023*

Lang, F. R., John, D., Lüdtke, O., Schupp, J. & Wagner, G. G. (2011), 'Short assessment of the big five: robust across survey methods except telephone interviewing', *Behavior Research Methods* **43**(2), 548–567.
URL: *https://doi.org/10.3758/s13428-011-0066-z*

Lazear, E. P. (2000), 'Performance pay and productivity', *American Economic Review* **90**(5), 1346–1361.

McGraw, K. O. (1978), The detrimental effects of reward on performance: A literature review and a prediction model, *in* M. R. Lepper & D. Greene, eds, 'The Hidden Costs of Reward: New Perspectives on the Psychology of Human Motivation', Lawrence Erlbaum Associates, Hillsdale, NJ, pp. 33–60.

Nystrom, H. (1990), Organizational innovation, *in* M. West & J. Farr, eds, 'Innovation and Creativity at Work: Psychological and Organizational Strategies', Wiley, New York, pp. 143–162.

Rudis, E. (2004), *CEO Challenge 2004: Perspectives and Analysis*, The Conference Board, New York.

Schumpeter, J. (1942), 'Creative destruction', *Capitalism, socialism and democracy* **825**, 82–85.

Shalley, C. E., Gilson, L. L. & Blum, T. C. (2000), 'Matching creativity requirements and the work environment: Effects on satisfaction and intentions to leave', *The Academy of Management Journal* **43**(2), 215–223.

Unsworth, K. (2001), 'Unpacking creativity', *The Academy of Management Review* **26**(2), 289–297.

Appendix A: Formal hypotheses

4.5 Hypothesis 1

Endogenous variable: Explorative behavior.

4.5.1 Hypothesis 1a

t-test (two-sided):
$H_0 : \mu_{Control} - \mu_{Strategy} = 0$
$\mathbf{H_1 : \mu_{Control} - \mu_{Strategy} \neq 0}$
OLS:
$H_0 : \beta_1 = 0$
$\mathbf{H_1 : \beta_1 \neq 0}$
Wilcoxon-Mann-Whitney-U-test:
$H_0 : a = 0$
$\mathbf{H_1 : a \neq 0}$

4.5.2 Hypothesis 1b

t-test (two-sided):
$H_0 : \mu_{Control} - \mu_{Profit} = 0$
$\mathbf{H_1 : \mu_{Control} - \mu_{Profit} \neq 0}$
OLS:
$H_0 : \beta_1 = 0$
$\mathbf{H_1 : \beta_1 \neq 0}$
Wilcoxon-Mann-Whitney-U-test:
$H_0 : a = 0$
$\mathbf{H_1 : a \neq 0}$

4.6 Hypothesis 2

4.6.1 Hypothesis 2a

Endogenous variable: Attention to strategy.
t-test (two-sided):
$H_0 : \mu_{Control} - \mu_{Strategy} = 0$
$\mathbf{H_1 : \mu_{Control} - \mu_{Strategy} \neq 0}$
OLS:
$H_0 : \beta_1 = 0$
$\mathbf{H_1 : \beta_1 \neq 0}$
Wilcoxon-Mann-Whitney-U-test:
$H_0 : a = 0$
$\mathbf{H_1 : a \neq 0}$

4.6.2 Hypothesis 2b

Endogenous variable: Attention to profit.
t-test (two-sided):
$H_0 : \mu_{Control} - \mu_{Profit} = 0$
$\mathbf{H_1 : \mu_{Control} - \mu_{Profit} \neq 0}$
OLS:
$H_0 : \beta_1 = 0$
$\mathbf{H_1 : \beta_1 \neq 0}$
Wilcoxon-Mann-Whitney-U-test:
$H_0 : a = 0$
$\mathbf{H_1 : a \neq 0}$

4.7 Hypothesis 3

Endogenous variable: Explorative behavior.

4.7.1 Hypothesis 3a

OLS:
$H_0 : \beta_3 = 0$
$\mathbf{H_1 : \beta_3 \neq 0}$
Wilcoxon-Mann-Whitney-U-test:
$H_0 : a = 0$
$\mathbf{H_1 : a \neq 0}$

4.7.2 Hypothesis 3b

OLS:
$H_0 : \beta_3 = 0$
$\mathbf{H_1 : \beta_3 \neq 0}$
Wilcoxon-Mann-Whitney-U-test:
$H_0 : a = 0$
$\mathbf{H_1 : a \neq 0}$

4.7.3 Hypothesis 3c

OLS (Dummy=1 if assigned to Profit):
$H_0 : \beta_3 = 0$
$\mathbf{H_1 : \beta_3 \neq 0}$
Wilcoxon-Mann-Whitney-U-test:
$H_0 : a = 0$
$\mathbf{H_1 : a \neq 0}$

Appendix B: Power Analysis

In the original paper of EM, subjects in the pay-for-performance contract yield on average a profit in the last period of 111 thalers. Because our control group is basically a copy of and mimics one-to-one the PfP group in EM, we assume that our control group will yield 111 thalers in the last period, too. Furthermore, based on a small test conducted in September

2019, we expect a standard deviation of $SD = 40$. It is our best assumption that the SD being equal for all three groups.

Next, we define the smallest detectable effect size of interest. Based on practical limitations, namely budget restrictions, we are willing to collect at most 300 observations in total (over all three groups, thus maximal 100 observations per group). To identify an effect with 80% power when comparing the control with one of the two treatment groups, with one interim look at the data, the effect must be at least 15.5 thalers large, translating in a Cohen's d of $d_s = 0.3875$. We deem such an effect also from a real-life perspective as appropriate - implementing a new reporting policy comes along with costs, and thus, the beneficial or detrimental effect should be large enough to be of practical relevance.

Next, we elaborate on the expected effect. In EM, the exploration contract yields in the final period on average a profit of 140. This leads to a profit difference between PfP and Exploration contract of $29(= 140 - 111)$. With a $SD = 40$, this yields a Cohen's d of $d_s = 0.725$. Most probably, our effects will be lower since our treatment interventions are based on a behavioural mechanism but do not change monetary incentives, as this is the case in EM. As a best-estimate, we expect our effect to be 30% (or 9 thalers) lower than in EM. Therefore, we estimate that subjects in the strategy treatment will yield a lower profit in the last period than the exploration contract of EM, and we estimate this to be at 131. This yields an effect size of 20(=131-111), or a Cohen's $d_s = 0.50$. For the profit treatment, we cannot base our estimates on a previous study due the lack of comparable alternatives. However, we expect the profit treatment to perform similarly as the strategy treatment (but in the opposite direction, of course). Consequently, we adopt the same Cohen's $d_s = 0.50$ for the profit treatment. Based on the expected effect size of d=.5, with power 80% and an alpha of 0.05, we obtain a sample size n=60 per group or n=180 in total (after both looks). We will have a first look at time =0.5, that is when 90 subjects are collected.

For controlling type 1 error rates, we use a linear spending function (power family function), as outlined in Jennison & Turnbull (2001). The alpha of 0.05 for a single look is adjusted for sequential analyses, namely for two looks using a linear spending function, yielding a nominal alpha of 0.0586.

Thus, when looking twice at the data, that is with one interim analyses, we formulate the analysis plan outlined in the main body of the text.

Appendix C: Variable definition

Variable (oTree def)	Elicited in	Group	Description	Properties	Purpose	Hypothesis
LS_treatment	Exp.	D	Treatment indicator	categorical: C, O, P	exogenous variation	TODO
LS_overallprofit	Exp.	Y	sum of total profit of all 20 periods	continuous, min:0 max:3982	outcome variable	none
LS_finloc	Exp	Y	final location	categorical: School, Business, Stadium	outcome variable	none
LS_finprofit	Exp	Y	profit in final round	continuous, min:0 max: 199.1	outcome variable	none
LS_maxprofit	Exp	Y	highest profit in all rounds	continuous, min:0 max: 199.1	outcome variable	none
LS_maxexpphase	Exp (constructed)	Y	Longest duration of an exploration phase. An exploration phase starts when subjects choose a location other than the default location suggested by the previous manager. An explorative phase is defined as ending when a subject switches back to the default location or when a subject does not change location and lemonade color and also does not change lemon content, sugar content and price by more than 0.25 units. Adapted 1:1 from EM.	discrete, min:0 max: 20	outcome variable	none
LS_durexpphase	Exp (constructed)	Y	Total duration of all exploration phases.	discrete, min:0 max: 20	outcome variable	none
LS_locnotdefault	Exp (constructed)	Y	Count of chosen non-default locations, i.e. non-Business locations.	discrete, min:0 max: 20	outcome variable	none
LS_stdvsugar	Exp (constructed)	Y	Standard deviation for sugar choices over all rounds	cont.	outcome variable	none
LS_stdvlemon	Exp (constructed)	Y	Standard deviation for lemon choices over all rounds	cont.	outcome variable	none
LS_stdvprice	Exp (constructed)	Y	Standard deviation for price choices over all rounds	cont.	outcome variable	none
LS_stdv3con	Exp (constructed)	Y	The average subject-specific standard deviation of strategy choices for the three continuous variables sugar, price, lemon	cont.	outcome variable	none
LS_report	Exp	CV	Reported text after every forth round (for the treatment groups only).	String	CV	none
LS_reportlength	Exp (constructed)	CV	Total length (in characters) of reported texts	discrete, min: 4 max: not limited	CV	none
LS_Dtotaltime	Exp	Y	Time elapsed for all 20 Decision-Screens.	cont.	outcome variable (effort proxy)	none
LS_Dtotalfocustime	Exp	Y	Focus time elapsed for all 20 Decision-Screens.	cont.	outcome variable (effort proxy)	none
LS_Dtotalunfocustime	Exp	CV	Unfocus time elapsed for all 20 Decision-Screens.	cont.	CV	none
LS_Dtotalfocusevents	Exp	CV	Sum of focus events for all 20 Decision-Screens.	discrete	CV	none
LS_Rtotaltime	Exp	Y	Time elapsed for all 20 Result-Screens.	cont.	outcome variable (effort proxy)	none
LS_Rtotalfocustime	Exp	Y	Focus time elapsed for all 20 Result-Screens.	cont.	outcome variable (effort proxy)	none
LS_Rtotalunfocustime	Exp	CV	Unfocus time elapsed for all 20 Result-Screens.	cont.	CV	none
LS_Rtotalfocusevents	Exp	CV	Sum of focus events for all 20 Result-Screens.	discrete	CV	none
LS_YEtotaltime	Exp	Y	Time elapsed for all 20 Reporting-Screens. Treatment groups only.	cont.	outcome variable (effort proxy)	none
LS_YEtotalfocustime	Exp	Y	Focus time elapsed for all 20 Reporting-Screens. Treatment groups only.	cont.	outcome variable (effort proxy)	none
LS_YEtotalunfocustime	Exp	CV	Unfocus time elapsed for all 20 Reporting-Screens. Treatment groups only.	cont.	CV	none
LS_YEtotalfocusevents	Exp	CV	Sum of focus events for all 20 Reporting-Screens. Treatment groups only.	discrete	CV	none

Table 3: Summary Of Variables, Experimental Task

Variable (oTree def)	Elicited in Module	Group	Description	Properties	Purpose	Hypothesis
openness	Big-5	μ	Openness score (Big5), between 1 and 7	categorical (ordinal)	μ	none
conscientiousness	Big-5	μ	Conscientiousness score (Big5), between 1 and 7	categorical (ordinal)	CV	none
extroversion	Big-5	μ	Extroversion score (Big5), between 1 and 7	categorical (ordinal)	CV	none
agreeableness	Big-5	μ	Extroversion score (Big5), between 1 and 7	categorical (ordinal)	CV	none
neuroticism	Big-5	μ	Extroversion score (Big5), between 1 and 7	categorical (ordinal)	CV	none
bigfivetime	Big-5	μ	Time elapsed for Big5 elicitation module	cont	aux	none
bigfivefocus	Big-5	μ	Focus time elapsed for Big5 elicitation module	cont	aux	none
bigfiveunfocus	Big-5	μ	Unfocus time elapsed for Big5 elicitation module	cont	aux	none
bigfivefocusevent	Big-5	μ	Number of focus off events for Big5 elicitation module	discrete	aux	none
q1	risk	λ	Risk. Self-assessment: Willingness to take risks in general."Please tell us, in general, how willing or unwilling you are to take risks."	categorical (ordinal)	auxiliary variable	none
riskc	risk	λ	Risk taking. Lottery choice sequence using staircase method	categorical (ordinal)	auxiliary variable	none
GPStime	risk	λ	Time elapsed for Risk preference elicitation module	cont	aux	none
GPSfocus	risk	λ	Focus time elapsed for Risk preference elicitation module	cont	aux	none
GPSunfocus	risk	λ	Unfocus time elapsed for Risk preference elicitation module	cont	aux	none
GPSfocusevent	risk	λ	Number of focus off events for Risk preference elicitation module	discrete	aux	none
			following two variables need to be constructed after the experiment, since it depends on other players data (zriskc indicated z-score of riskc)			
GPSrisk	risk (constructed)	λ	0.4729985 x zriskc + 0.5270015 x zq1	cont (z-score)	CV	3

Table 4: Summary Of Variables, Modules after experimental task
